# In Vitro and In Vivo Radiotoxicity and Biodistribution of Thallium-201 Delivered to Cancer Cells by Prussian Blue Nanoparticles

**DOI:** 10.1021/acsami.4c21700

**Published:** 2025-02-21

**Authors:** Katarzyna M. Wulfmeier, Juan Pellico, Pedro Machado, M. Alejandra Carbajal, Saskia E. Bakker, Rafael T. M. de Rosales, Kavitha Sunassee, Philip J. Blower, Vincenzo Abbate, Samantha Y. A. Terry

**Affiliations:** † School of Biomedical Engineering and Imaging Sciences, King’s College London, London SE1 7EH, U.K.; ‡ Centre for Ultrastructural Imaging, King’s College London, London SE1 9RT, U.K.; § Oxford Instruments NanoAnalysis, High Wycombe HP12 3SE, U.K.; ∥ Advanced Bioimaging, University of Warwick, Coventry CV4 7AL, U.K.; ⊥ Institute of Pharmaceutical Sciences, King’s College London, London SE1 9NH, U.K.

**Keywords:** ^201^Tl, Prussian blue nanoparticles, thallium binding, targeted radionuclide therapy, Auger electron-emitters

## Abstract

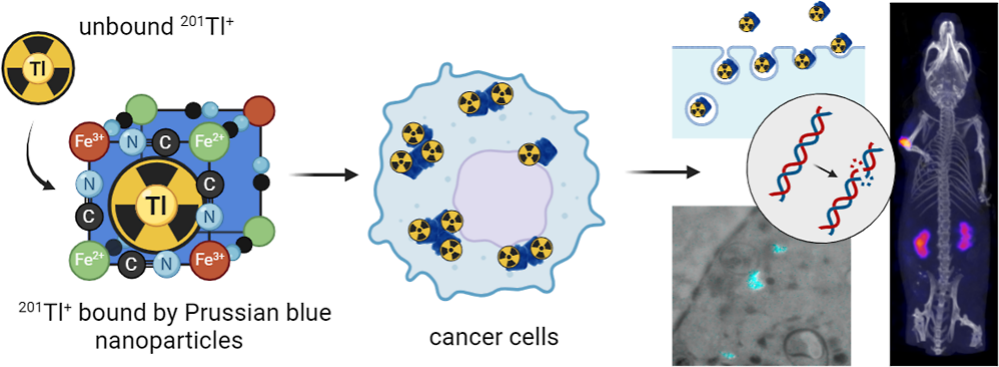

Thallium-201 (*t*_1/2_ = 73 h) emits around 37 Auger and other secondary electrons per decay and is highly radiotoxic when internalized into cancer cells. However, the lack of effective chelators hinders its application in molecular radiotherapy. This study evaluates Prussian blue nanoparticles, coated with citric acid (^201^Tl-caPBNPs) or chitosan (^201^Tl-chPBNPs), as a ^201^Tl delivery vehicle compared with unbound ^201^Tl^+^. Cellular uptake and efflux kinetics and radiotoxicity using clonogenic and γH2AX DNA damage assays were evaluated in vitro for both nanoparticle types. Subcellular localization was also assessed using electron microscopy with energy-dispersive X-ray spectroscopy. Biodistribution of ^201^Tl-chPBNPs was evaluated in vivo in mice bearing subcutaneous A549 tumor xenografts, using single photon computed tomography imaging and ex vivo tissue counting. Compared with unbound ^201^Tl^+^, ^201^Tl-chPBNPs showed higher cellular uptake, while ^201^Tl-caPBNP uptake was reduced. Both showed delayed efflux of ^201^Tl from cancer cells. PBNPs prelocalized within cells enhanced the capture and retention of ^201^Tl^+^ ions. Both types of PBNPs accumulated in cytoplasmic vesicular compartments and were not visible in the nuclei. Furthermore, ^201^Tl-radiolabeled chPBNPs but not ^201^Tl-caPBNPs showed significantly greater radiotoxicity than unbound ^201^Tl^+^ per Becquerel of radiotoxicity provided in media, resulting from their higher uptake and delayed efflux. However, when corrected for the greater activity accumulated in cells and delayed efflux, the radiotoxicity of ^201^Tl-chPBNPs was lower than that of unbound ^201^Tl^+^, possibly due to differences in subcellular localization. These findings highlight the potential of chPBNPs for enhancing the uptake and retention of ^201^Tl in cancer cells and development of targeted radionuclide therapy.

## Introduction

Radionuclides emitting short-range, low-energy Auger electrons are of particular interest for radiotherapy, allowing precise targeting of cancer cells, including micrometastases and circulating tumor cells.^1^ Among the Auger electron emitters, ^201^Tl has been identified as one of the most promising radionuclides for targeted radiotherapy.^2^ It has one of the highest numbers of Auger and other secondary electrons emitted per decay (average 36.9),^3^ a convenient half-life (73 h), a stable daughter product (^201^Hg), and worldwide availability due to its previous extensive widespread use in myocardial imaging.^4^ Additionally, it exhibits adequate molar activity and favorable cellular dosimetry.^2,5^ Indeed, our preliminary in vitro studies demonstrated the significant radiotoxic potential of internalized ^201^Tl, leading to increased nuclear DNA damage and clonogenic toxicity compared to clinically evaluated Auger electron-emitters such as ^111^In and ^67^Ga.^6−8^ However, a major hurdle identified for therapeutic applications of ^201^Tl is the lack of effective chelators with which to incorporate it into tumor-selective targeting vehicles.^2^

Efforts have previously been made to chelate ^201^Tl in the 1+ and 3+ oxidation states. Kryptofix derivatives were evaluated as chelators for [^201^Tl]Tl^+^ to selectively deliver it to prostate cancer cells upon conjugation with a PSMA-targeting motif; while they exhibited high radiolabeling yield and in vitro stability in serum, both alone and in the conjugates, incubation with cells resulted in the release of Tl^+^ from the complexes.^9^ A range of commonly-used chelators, including DOTA, DTPA, EDTA, and picolinic acid derivatives, were evaluated for binding [^201^Tl]Tl^3+^, but the complexes were found to be unstable, likely due to the easy and quick reduction of Tl^3+^ to the 1+ oxidation state, which results in metal dissociation from the complex.^10−12^

A substance known for its ability to bind thallium(I) is Prussian blue [PB, ferric hexacyanoferrate(II), (Fe^(III)^_4_[Fe^(II)^(CN)_6_]_3_·*x*H_2_O)], which is registered by the US Food and Drug Administration (FDA) for treating radioactive/nonradioactive thallium and radioactive cesium poisoning.^13^ It is a dark blue pigment that forms a cubic crystalline structure composed of Fe^2+^ and Fe^3+^ linked through coordinated cyanide groups. It exhibits high affinity for binding monovalent thallium ions (Tl^+^) within the interstitial spaces created by the lattice structure.^14^ Recently, PB has regained attention from the scientific community, as it can be easily assembled into a diverse range of nanoparticles (PBNPs) of different shapes and sizes with unique and tunable physicochemical properties,^15,16^ leading to wide-ranging applications as ion exchange materials, ion batteries, photomagnets, electrochemical sensors, and biosensors.^17−21^ Moreover, the water dispersibility and biocompatibility of PBNPs have encouraged exploration of their uses in biomedical research. For example, PBNPs have served as drug carriers, contrast agents for magnetic resonance imaging and photoacoustic imaging, nanoenzymes, and sensitizers for photothermal therapy.^22−28^ Ultrasmall PBNPs, coated with aminopolyethylene glycol or glucose-functionalized aminotriethylene glycol to control their biodistribution, have been used for ^201^Tl delivery for diagnostic imaging. They showed in vivo accumulation of ^201^Tl-PBNPs in lungs and liver during the first 3 h after intravenous (i.v) injection, whereas unbound ^201^Tl^+^ at these time points accumulated mainly in kidneys.^29^^201^Tl-radiolabeled citric acid-coated PBNPs have been investigated as contrast agents for dual single-photon emission computed tomography and magnetic resonance imaging (SPECT/MRI).^30^ In another study, different core shapes and coatings (including dextran and a phospholipid bilayer) were shown by SPECT to control the biodistribution of the PBNPs in vivo.^31^ Despite these uses of ^201^Tl-PBNPs in preclinical nuclear imaging, their radiotoxic effects in cancer radionuclide therapy remain unexplored.

We have previously described the synthesis and physicochemical characterization of two types of PB nanoparticles (PBNPs), coated with citric acid (caPBNPs) and chitosan (chPBNPs).^14^ These coatings imparted distinct shapes and opposite surface charges (negative and positive, respectively). Both types of PBNPs proved to efficiently bind ^201^Tl^+^ with high radiochemical stability for at least 72 h under physiological conditions. Furthermore, it was experimentally confirmed that the thallium inclusion mechanism involves thallium ions occupying interstitial spaces within the PB crystal structure, affecting the ionic composition but not the structure of the core or shell of the nanoparticles.^14^ In the present study, we compared the in vitro accumulation and retention of ^201^Tl within cancer cells, as well as the subcellular localization and radiotoxicity of ^201^Tl, when administered in the form of ^201^Tl-caPBNPs, ^201^Tl-chPBNPs, and unbound ^201^Tl^+^. We also evaluated the ability of unlabeled intracellular PBNPs to sequester ^201^Tl administered to cells in the form of free ^201^Tl^+^ ions. Finally, the in vivo and ex vivo biodistribution and retention of radiolabeled chPBNPs were evaluated after direct injection into subcutaneous tumor xenografts in mice and compared to unbound ^201^Tl^+^.

## Methods and Materials

Cell culture consumables and chemicals, unless otherwise specified, were purchased from Sigma-Aldrich, UK. [^201^Tl]TlCl in sterile 0.9% NaCl solution (280–580 MBq/5.8 mL) was obtained from Curium Pharma, France.

### Synthesis of PB Nanoparticles

The synthesis of citric acid-coated PB nanoparticles (caPBNPs) and chitosan-coated PB nanoparticles (chPBNPs) was described previously.^14^ Briefly, for caPBNPs, 105 mg of citric acid (as monohydrate, 0.5 mmol) was added to 20 mL of 1 mM FeCl_3_. The solution was heated to 60–65 °C, and then 20 mL of 1 mM K_4_[Fe(CN)_6_]·3H_2_O (Alfa Aesar) with 105 mg of citric acid was added dropwise over 10 min.^30^ The suspension was left for another minute stirring at 60–65 °C and then cooled to room temperature (RT). To purify the nanoparticles, 40 mL of the caPBNPs suspension was transferred to Amicon filter tubes (15 mL, 30,000 MWCO) and centrifuged at 4200 rpm for 10 min (Rotina 380R, Hettich). The filtrate solution was discarded, and caPBNPs were resuspended with Milli-Q water and centrifuged again. This process was repeated twice. CaPBNPs were resuspended in 5 mL of Milli-Q water (to a concentration of approximately 1 mg/mL); pH: 6–7. To obtain dry powder, the aqueous suspension of caPBNPs was freeze-dried at −54 °C, 0.1 mbar (LTE Scientific Freeze-Dryer Lyotrap).

The synthesis of chPBNPs was based on a previously published method.^32^ A chitosan solution (0.1 mg/mL in 0.5 M HCl) was stirred for 1 h at RT and filtered through a 0.45 μm filter. Then, 5 mL of a 1 mM K_3_Fe(CN)_6_ aqueous solution was added to 20 mL of the chitosan solution at RT while stirring. After 30 min, 5 mL of 1 mM FeCl_2_·4H_2_O was added dropwise, and the mixture was stirred for 1 h at RT. Finally, 50 mL of acetone was added, and particles were collected by centrifugation at 4000 rpm for 10 min, washed with a mixture of 0.5 M HCl and acetone (20:80 v/v) three times, collected by centrifugation, and dried under vacuum for 24 h.

### Incubation and Physicochemical Characterization of PB Nanoparticles with Nonradioactive Thallium

2.5 mL of either caPBNPs or chPBNPs suspension in water (typical concentration: 1 and 0.5 mg/mL, respectively) was added to 2.5 mL of TlCl aqueous solution (2000 mg/L) and incubated at RT for 3 h. The resulting suspension of Tl-PBNPs was purified by ultrafiltration using Amicon filter tubes (0.5 mL 10,000 MWCO). The mixture was centrifuged at 13,800*g* for 5 min and the pellet resuspended in H_2_O. This process was repeated three times. Finally, in order to obtain the dry powder, the aqueous suspension of Tl-PBNPs was freeze-dried at −54 °C, 0.1 mbar (LTE Scientific Freeze-Dryer Lyotrap). Physicochemical characterization of caPBNPs and chPBNPs, including the radiolabeling efficiency and stability with ^201^Tl was described previously.^14^

### Cell Culture

Human breast adenocarcinoma cells MDA-MB-231 (ATCC HTB-26) were cultured in Dulbecco’s Modified Eagle Medium (DMEM, low glucose 1000 mg/L). Human prostate cancer cells DU145 (ATCC HTB-81), human lung cancer cells A549 (ATCC CCL-185), and human ovarian cancer cells SKOV3 (ATCC HTB-77) were cultured in RPMI-1640 medium. Both media were supplemented with 10% fetal bovine serum, 5% l-glutamine, penicillin (100 units), and 100 μg/mL streptomycin. Cultured cells were trypsinised and seeded at 250,000 cells per well for all experiments in 24-well plates 16 h before each experiment and grown at 37 °C in a humidified 5% CO_2_ atmosphere. 25 mM KCl (BDH Laboratory) was used to inhibit unbound ^201^Tl uptake.^6^ Monthly testing ensured that cells were mycoplasma-negative (Eurofins).

### Cellular Uptake and Efflux

Cells were prepared in multiwell plates as described above. Fifteen minutes before each experiment, the medium in each well was replaced by 200 μL of fresh medium. To assess how K^+^ affects the uptake of Tl-PBNPs, certain cells were incubated with 25 mM KCl. To measure ^201^Tl uptake, stock [^201^Tl]TlCl solution or ^201^Tl-caPBNPs/^201^Tl-chPBNPs were diluted with water to the required concentration (0.6–20 kBq/μL), and 50 μL was added to each well. CaPBNPs were used within a concentration range of 0.1–0.4 mg/mL, while the concentration of chPBNPs was 0.05 mg/mL. Plates were incubated at 37 °C for a duration ranging from 1.5 to 6 h. At the end of each experiment, cells were manually counted using a hemocytometer. Then, the radioactive incubation solution was collected; adherent cells were briefly washed thrice with PBS and lysed with 1 M NaOH for 15 min at RT. Unbound radioactivity (incubation medium and PBS washings) and cell-bound radioactivity (lysate) were measured with a CompuGamma CS1282 gamma counter. At the end of each experiment, empty plates showed minimal residual activity attached to the plastic.

To measure the rate of cellular efflux of ^201^Tl or ^201^Tl-caPBNPs/^201^Tl-chPBNPs, cells were first incubated at 37 °C for 3 h with 50 kBq of ^201^Tl or ^201^Tl-PBNPs in 250 μL of medium, which was then removed. Adherent cells were washed briefly with 250 μL PBS, and 250 μL of fresh nonradioactive medium was added to each well. Cells were incubated for 15–180 min at 37 °C, after which medium was collected, and the cells were washed and lysed. The activity inside cells was measured as described above.

The “continuous” cellular efflux experiment involved repeated medium changes on the same cells. In brief, after incubation with 30 kBq [^201^Tl]TlCl, ^201^Tl-caPBNPs, or ^201^Tl-chPBNPs as above, replacing the radioactive medium with fresh medium and allowing a further 15 min of efflux, the medium was again removed, cells were washed once with PBS, and fresh medium added. This process was repeated 4 times at intervals from 15 to 60 min. After 180 min, cells were lysed and ^201^Tl activity was measured as described above, converting CPM to activity by means of a calibration curve. The retained activity was expressed as a percentage of the activity found inside cells at each time point compared to the activity accumulated inside cells at the beginning of the experiment.

To compare ^201^Tl uptake and efflux in cancer cells with and without prior incubation with PBNPs, 50 μL of caPBNPs (1 or 0.5 mg/mL) or chPBNPs (0.25 mg/mL) was added to A549 cells in 200 μL of medium and incubated for 16 h. The cells were then washed thrice with PBS. 200 μL of fresh medium and 50 μL of stock [^201^Tl]TlCl (50 kBq) were added and incubated at 37 °C for 90 min. After this time, the protocols for uptake and efflux described earlier were followed.

### Radiotoxicity

Clonogenic assays were carried out to assess the reproductive clonogenicity of A549 cancer cells after exposure to ^201^Tl, ^201^Tl-caPBNPs, and ^201^Tl-chPBNPs and nonradioactive caPBNPs and chPBNPs. 50 μL of 250–1000 kBq [^201^Tl]TlCl, ^201^Tl-caPBNPs/^201^Tl-chPBNPs, or nonradioactive PBNPs were added to each well containing 250,000 A549 cells in 200 μL medium. The concentration of caPBNPs and chPBNPs used was 0.1 and 0.05 mg/mL, respectively. Some wells were incubated with 25 mM KCl. Additional wells replicating each incubation were used for uptake measurements, as described above. After 3 h, the radioactive incubation solution was removed, and cells were washed thrice with PBS, trypsinised, resuspended in nonradioactive medium, seeded at 1000 cells/well in a 6-well plate, and cultured for 5–8 days, changing medium every 2–3 days. Colonies were fixed and stained with 0.05% crystal violet in 50% methanol and counted manually, defining colonies as containing >50 cells. Curves showing survival as a function of radioactivity added to each well were generated, and the survival data were combined with simultaneously acquired uptake data to generate curves showing survival as a function of average accumulated activity per cell. These data were fitted to a linear quadratic survival model *Y* = 100 × exp(−1 × (*A* × *X* + *B* × X^2^)), using GraphPad Prism 10, to calculate *A*_50_ and *A*_10_ (average accumulated activity per cell necessary to achieve, respectively, 50 and 90% reduction in clonogenic survival) along with the corresponding 95% confidence intervals.

A γH2AX assay was used to estimate ^201^Tl-induced DNA damage. 250,000 A549 cells were seeded on coverslips coated with poly-*l*-lysine (50 μg/mL) placed in each well of a 24-well plate. Following a 3 h incubation with 50 μL of [^201^Tl]TlCl or ^201^Tl-caPBNPs/^201^Tl-chPBNPs solutions (1000–4000 kBq/mL), the concentrations of caPBNPs and chPBNPs used were 0.1 and 0.05 mg/mL, respectively. Medium was removed, and coverslips were washed with PBS and fixed with 3.7% formalin in PBS. The cells were then treated for 15 min with 0.5% Triton X-100 and 0.5% IGEPAL CA-630 solution, incubated with 1% goat serum/2% bovine serum albumin (BSA) in PBS for 1 h, washed with PBS, incubated overnight at 4 °C with mouse antiphospho-histone H2A.X monoclonal antibody (Merck, 1:1600 in 2% BSA), washed with 2% BSA in PBS, and incubated with goat antimouse secondary fluorescent antibody Alexa Fluor488 (Invitrogen, 1:500 in PBS) for 2 h at 4 °C. Cells were stained and mounted with Prolong Gold Antifade Reagent with DAPI (Invitrogen). A TCS SP5 confocal microscope with Leica software was used to obtain fluorescent images and CellProfiler was used to quantify numbers of γH2AX foci per nucleus.

### Energy-Dispersive X-ray Spectroscopy Combined with Transmission Electron Microscopy

Energy-dispersive X-ray spectroscopy (EDS) with transmission electron microscopy (TEM) was performed at the Centre for Ultrastructural Imaging, KCL. Sample preparation involved a carbon-coated sapphire disc (Leica ACE 600, Leica Microsystems) placed in each well of a 24-well plate and incubated with poly-l-lysine (50 μg/mL) for 30 min 75,000 lung cancer cells (A549) were seeded, and 15 min before the experiment, the medium in each well was replaced by 200 μL of fresh medium and 50 μL of either caPBNPs, Tl-caPBNPs (final concentration: 0.1 mg/mL), chPBNPs or Tl-chPBNPs (final concentration: 0.05 mg/mL) was added into each well. After 3 h incubation time, medium was removed, and the sapphires were high-pressure-frozen using a Leica EM ICE (Leica Microsystems, Vienna) and subsequently freeze-substituted (Leica AFS 2, Leica Microsystems, Vienna) with 0.2% uranyl acetate in dry acetone and finally embedded into Lowicryl HM20. Thin sections were cut using an ultramicrotome (UC7, Leica Microsystems) and collected on carbon support film GS 2 × 1 copper grids (TAAB Laboratories, UK). All experiments were performed on a JEOL JEM F200 STEM instrument (JEOL, Japan) operated at 200 kV in STEM mode, using a standard TEM holder. The holder was tilted 19° toward the Ultim Max EDS detector (Oxford Instruments). The EDS data sets were analyzed using AZtec version 6.1. To determine the concentration of elements present in the samples, QuantMaps were generated for quantitative analysis. Comparative spectra were collected from selected regions to analyze the differences between regions with and without nanoparticles. The amount of thallium within PBNPs was expressed as the percentage of total weight (wt %), and the average was calculated based on the data obtained from 10 energy spectra of the regions containing Tl-PBNPs and cytoplasmic regions without visible nanoparticles in various cells and distant locations. EDS maps and TEM overlays were generated to visualize the elemental distribution across different imaging sites.

### In Vivo Study with chPBNPs

chPBNPs were synthesized as described earlier and radiolabeled with [^201^Tl]TlCl at 70 °C for 1 h (final chPBNPs concentration: 0.25 mg/mL, activity concentration: 43 MBq/mL, radiolabeling efficiency: 95.3 ± 0.6%). [^201^Tl]TlCl for the control group injected with unbound thallium was obtained by diluting the stock solution with 0.9% NaCl to the required activity concentration. Cell culture and tumor inoculation: human lung cancer cells (A549) were cultured and harvested as described earlier. The cell suspension in PBS was prepared at a concentration of 20 × 10^6^ cells/mL. Animal experiments were performed in accordance with the Animals (Scientific Procedures) Act 1986, with protocols approved by UK Home Office and King’s College London animal welfare and ethical review body (St Thomas’ Campus) under Home Office Project Licenses. Ten female BALB/C nu/nu mice (8 weeks old) were purchased from Charles River Laboratories (UK). After 1 week of acclimatization, the animals were subcutaneously injected with 2 × 10^6^ A549 cells (100 μL) in the left flank below the shoulder (under brief anesthesia: 2.0% isoflurane, O_2_ flow rate of 1.0 L/min) and monitored for the health status, weight, and tumor size. Tumor dimensions were measured by calliper and volume was calculated using the formula *V* = (*w*^2^ × *l*)/2 (where “*w*” is the tumor width and “l” is the length).^33^ The average tumor volume after 5 weeks was 252.7 ± 104.9 mm^3^ (*n* = 9).

### SPECT/CT Imaging

Mice were randomized across 2 groups: the control group, where 4 mice were injected with [^201^Tl]TlCl, and 5 mice injected with ^201^Tl-chPBNPs. SPECT/CT imaging was done for 3 mice in each group, followed by the ex vivo biodistribution study. The remaining mice were used for the ex vivo biodistribution study only. Mice were anesthetized with 2.0% isoflurane (VetTech Solutions Ltd.) at a O_2_ flow rate of 1.0 L/min, and 0.4–0.5 MBq of ^201^Tl in the required form in 10 μL volume was injected into the tumors. After 1 h under anesthesia, 3 mice from the group were imaged for 30 min with the SPECT/CT NanoScan 80 W (Mediso Ltd., Budapest, Hungary) with three-mouse hotel settings (see Supporting Information). After SPECT/CT scanning, mice were allowed to recover from anesthesia. All mice were reanesthetized and rescanned by SPECT/CT at 24 and 48 h post injection. When the 48 h scanning procedure was completed, mice were culled, and dissection of organs was performed immediately for the quantification of ex vivo biodistribution of ^201^Tl. VivoQuant v3.5-patch2 software (inviCRO, Massachusetts, USA) was used to view and quantify all reconstructed images. SPECT/CT biodistribution quantification: the regions of interest were manually contoured for tumors and kidneys using CT to mark their boundaries. SPECT/CT images were shown as maximum intensity projection (MIPs) or in transverse sections with the same intensity scale bar for all the groups. Data are expressed as standardized uptake values (SUV) and decay-corrected. For ex vivo biodistribution, organs and tissues were dissected and weighed. Radioactivity in each organ was then measured with the CompuGamma CS1282 gamma counter (over 60 s, energy window: 81–110) and converted to Bq using a [^201^Tl]TlCl calibration curve.^6^ Biodistribution data are expressed as percentage of injected activity (IA) (% IA, decay-corrected) or percentage of IA per gram (% IA/g, decay-corrected).

### Statistical Analysis

Data are expressed as mean ± SD. To compare two sets of measurements and assess the significance, two statistical tests were used: parametric *t*-test (where data were shown to be normally distributed) and nonparametric Mann–Whitney test (where data could not be shown to be normally distributed). Shapiro–Wilk test was used to check the measurements for normal distribution. A value of *P* < 0.05 was considered statistically significant. Figures were created with GraphPad Prism 10.

## Results

The synthesis, characterization, and radiolabeling of caPBNPs and chPBNPs were performed as previously reported.^14^ Negatively-charged caPBNPs had a regular cubic shape with a size of approximately 47 nm, while positively-charged chPBNPs were spherical and larger, with an average diameter of around 58 nm when measured by TEM. Radiolabeling of both types of PBNPs with [^201^Tl]TlCl was efficient, producing radiochemically stable ^201^Tl-caPBNPs and ^201^Tl-chPBNPs.^14^

**Figure 1:**
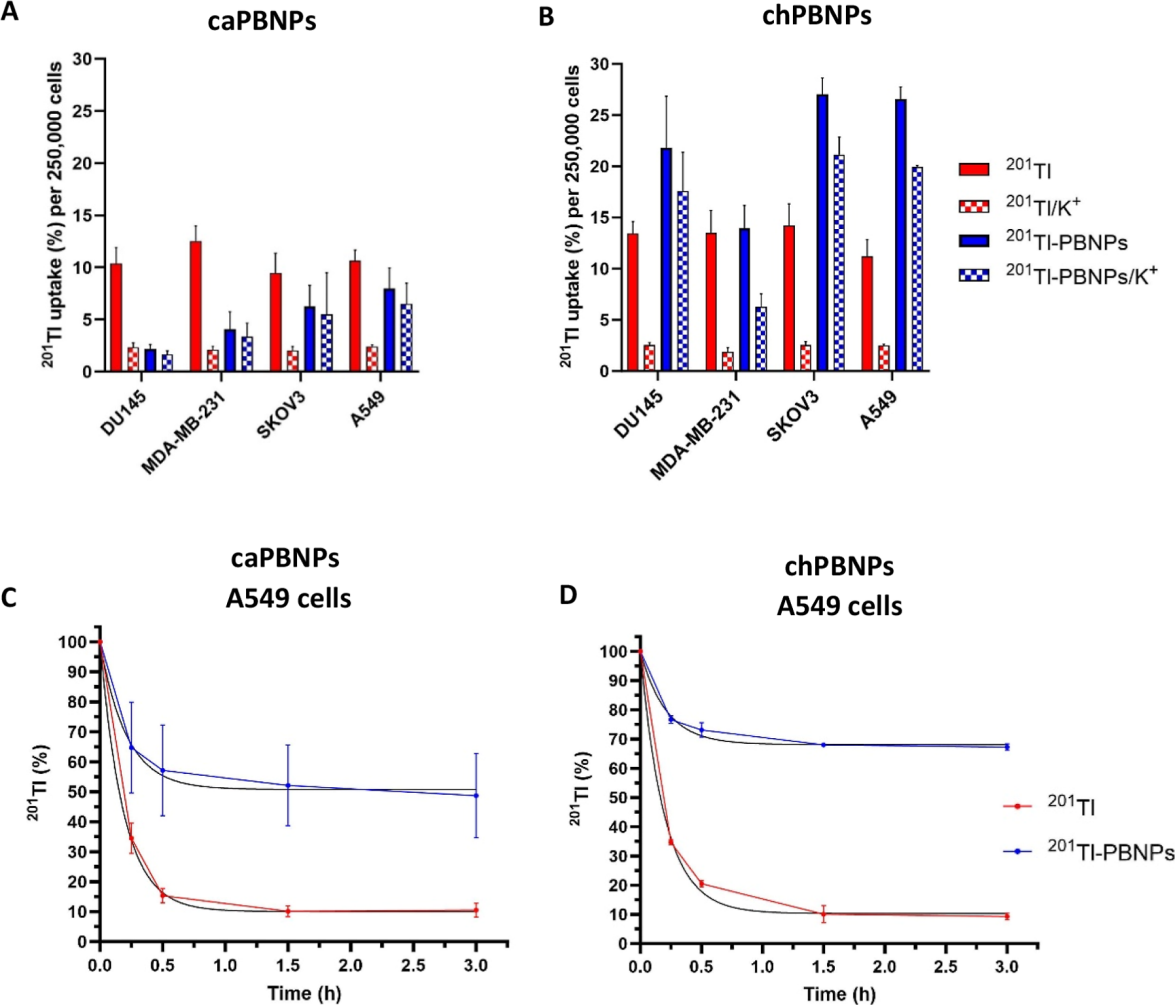
^201^Tl-PBNP uptake and efflux in cancer cells. (A) Uptake of ^201^Tl^+^ and ^201^Tl-caPBNPs in prostate cancer cells (DU145), breast cancer cells (MDA-MB-231), ovarian cancer cells (SKOV3), and lung cancer cells (A549) after 3 h incubation; (B) uptake of ^201^Tl^+^ and ^201^Tl-chPBNPs in DU145, MDA-MB-231, SKOV3, and A549 cells after 3 h incubation; (C) efflux of ^201^Tl^+^ and ^201^Tl-caPBNPs from A549 cells; (D) efflux of ^201^Tl^+^ and ^201^Tl-chPBNPs from A549 cells. The amount of activity in cells from the preceding 3 h uptake period is defined as 100% at time 0, at which time radioactive medium was replaced with nonradioactive medium and cell-bound ^201^Tl activity was measured over time. A nonlinear (exponential) regression line was fitted (black line, *Y* = (*Y*_0_ – plateau) × exp(−*K* × *X*) + plateau). Data are presented as mean ± SD, *n* = 3, triplicates. 25 mM KCl was used to inhibit unbound ^201^Tl^+^ uptake [(A,B), red and blue checked bars]. CaPBNP concentration in medium: 0.1 mg/mL; chPBNPs concentration in medium: 0.05 mg/mL. ^201^Tl activity used for all conditions: 30 kBq/well; 250,000 cells per well were seeded for each experiment.

Cellular uptake of ^201^Tl-caPBNPs and ^201^Tl-chPBNPs was measured after 3 h of incubation across different human cancer cells, namely, DU145 (prostate cancer), MDA-MB-231 (breast cancer), A549 (lung cancer), and SKOV3 (ovarian cancer) (Figure 1A,B); each cell line was chosen for its endocytic properties. KCl solution was also added to a group of samples to inhibit the uptake of unbound ^201^Tl^+^.^6^ Uptake of the negatively charged ^201^Tl-caPBNPs ranged between 2.2 ± 0.4% and 8.0 ± 2.0% per 250,000 cells; this is substantially lower than the uptake of unbound ^201^Tl^+^, which ranged between 9.5 ± 1.9% and 12.6 ± 1.4% (Figure 1A). Uptake of the positively charged ^201^Tl-chPBNPs, on the other hand, was on average 1.7 times higher than the uptake of unbound ^201^Tl^+^, ranging between 13.9 ± 2.2% and 27.1 ± 1.6% (Figure 1B). Potassium ions added to the medium dramatically reduced uptake of unbound ^201^Tl^+^ ions (typically by about 80%), while the suppression of uptake of ^201^Tl-caPBNPs and ^201^Tl-chPBNPs by KCl was relatively modest, typically about 18% and 30% respectively (Figure 1A,B). Lung cancer A549 cells were chosen as a model cell line for further experiments due to their high uptake rates of ^201^Tl-chPBNPs. Dynamic uptake measurements showed that whereas unbound ^201^Tl^+^ reached a plateau after 90 min (Figure S1A,B), ^201^Tl uptake from ^201^Tl-caPBNPs and ^201^Tl-chPBNPs in A549 cells continued to increase for at least 6 h. Overall, ^201^Tl-chPBNPs was taken up most efficiently than unbound ^201^Tl^+^, and the relatively marginal effect of added potassium on ^201^Tl-chPBNP and ^201^Tl-caPBNP uptake suggests that the principal uptake mechanism of PBNPs, unlike that of unbound ^201^Tl^+^, is unrelated to potassium transport.

Efflux assays were conducted to evaluate the rate at which intracellular radioactivity was eliminated from cells after a 3 h uptake period. Accumulated radioactivity was washed out from A549 cancer cells labeled with either ^201^Tl-caPBNPs or ^201^Tl-chPBNPs significantly more slowly than from cells labeled with unbound ^201^Tl^+^; after 1 h of efflux, around 50.8% (95% confidence interval (Cl): 43.8–57.3%) remained in cells in the case of ^201^Tl-caPBNPs compared to 10.0% (95% Cl: 7.5–12.6%) for unbound ^201^Tl^+^ (Figure 1C). ^201^Tl-chPBNPs showed an even lower wash out rate, leveling at around 68.1% (95% Cl: 62.7–73.3%; Figure 1D) after 1 h of efflux. With repeated changes of surrounding medium designed to mimic the physiological environment of perfused tumors, 50% of the initial activity of ^201^Tl-caPBNPs was washed out within 0.34 h, stabilizing at 23.4% by the end of the experiment, whereas unbound ^201^Tl was almost completely washed out within 0.13 h (Figure S1C). Under these conditions, it took nearly 1 h for 50% of the initially accumulated activity of ^201^Tl-chPBNPs to wash out, reaching a steady state of 39.3% at the end of the experiment (Figure S1D). Thus, PBNPs significantly reduce the rate of efflux compared with unbound thallium.

**Figure 2:**
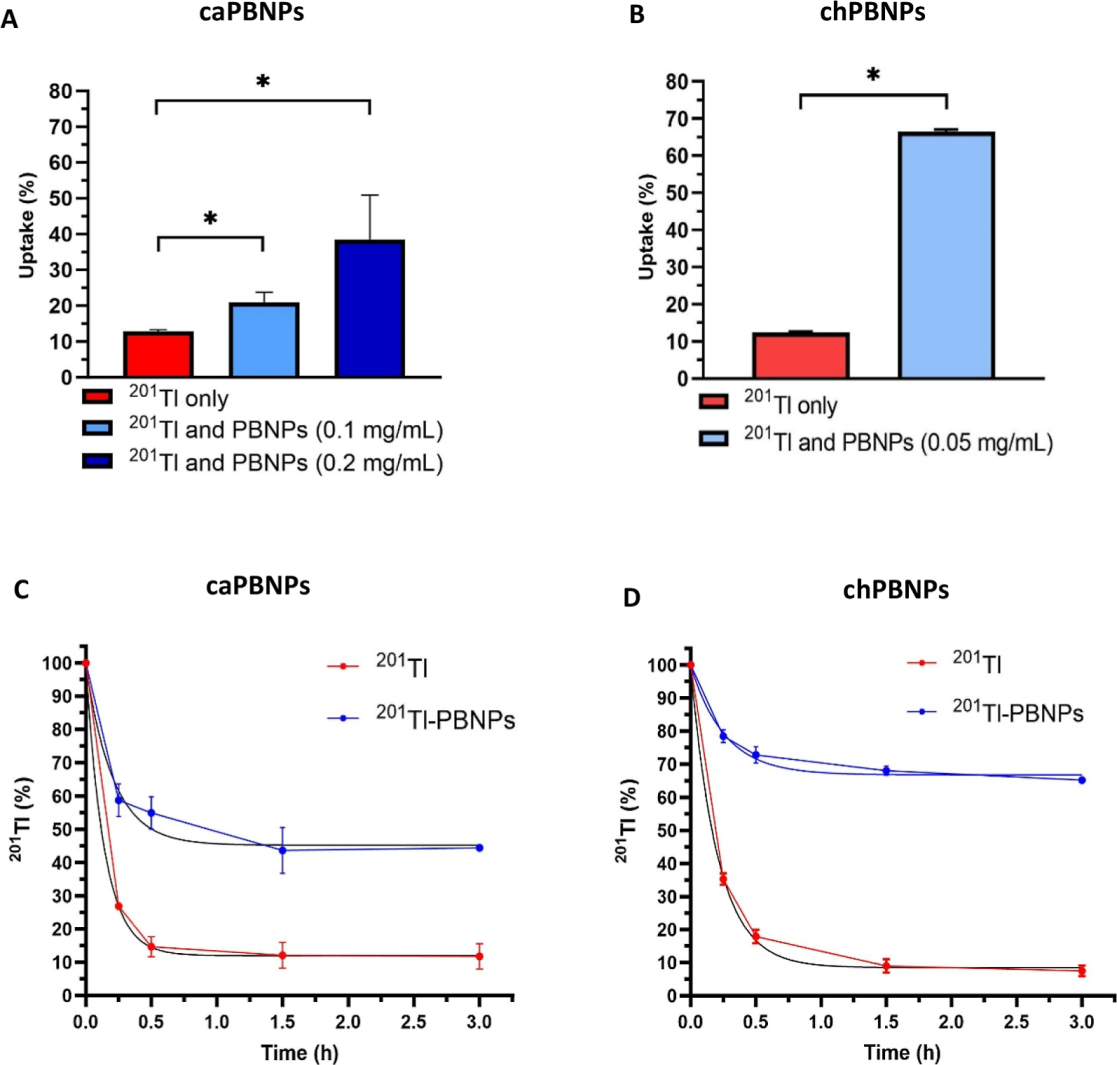
^201^Tl^+^ uptake and retention in A549 lung cancer cells preincubated with unlabeled caPBNPs and chPBNPs. (A) ^201^Tl^+^ uptake after 90 min incubation with 30 kBq/well [^201^Tl]TlCl in lung cancer cells (A549) where caPBNPs are absent (red bar) and preincubated with caPBNPs (blue bars). Concentration of caPBNPs in the medium: 0.1 and 0.2 mg/mL; (B) ^201^Tl^+^ uptake after 90 min incubation with 30 kBq/well [^201^Tl]TlCl in A549 where chPBNPs are absent (red bar) and preincubated with chPBNPs (blue bar). Concentration of chPBNPs in the medium: 0.05 mg/mL; (C) percentage of ^201^Tl activity remaining in A549 cells preincubated with 0.1 mg/mL of caPBNPs, followed by incubation with [^201^Tl]TlCl; (D) percentage of ^201^Tl activity remaining in A549 cells preincubated with 0.05 mg/mL of chPBNPs, followed by incubation with [^201^Tl]TlCl. The amount of activity from the preceding uptake period is defined as 100% at time 0, at which radioactive medium was replaced with nonradioactive medium, and retained ^201^Tl activity was measured over time (vertical axis). A nonlinear regression exponential line was fitted (black line, *Y* = (*Y*_0_ – plateau) × exp(−*K* × *X*) + plateau). Data are presented as mean ± SD, triplicates, *n* = 3, 250,000 cells per well were seeded for each experiment. * indicates significance with *P* < 0.05, unpaired *t*-test.

To assess the ability of intracellular caPBNPs and chPBNPs to capture radioactive thallium administered as ^201^Tl^+^, A549 cells were preincubated with unlabeled caPBNPs or chPBNPs for 16 h. Noninternalized PBNPs were then removed with the medium, and cells were washed and incubated for 1.5 h with [^201^Tl]TlCl. In cells preincubated with caPBNPs, [^201^Tl]TlCl uptake was significantly higher compared to cells without prior incubation with nanoparticles, and ^201^Tl uptake increased with the concentration of nanoparticles initially added to the well (Figure 2A). The chPBNPs proved to be even more effective than caPBNPs at capturing ^201^Tl^+^ in this manner; ^201^Tl^+^ uptake after 1.5 h incubation increased from 12.4 ± 0.2% in untreated cells to 66.4 ± 0.7% in cells that had been preincubated with chPBNPs at a concentration of 0.05 mg/mL (Figure 2B). Moreover, ^201^Tl washout from cells containing caPBNPs or chPBNPs was significantly delayed compared to washout from untreated cells, leveling at 45.2% (95% Cl: 31.9–57.2) and 66.8% (95% Cl: 61.1–72.0), respectively (Figure 2C,D), after a 3 h efflux period, compared to ca. 10% for untreated cells. These experiments proved that PBNPs not only are efficient at delivering radioactive thallium to cancer cells but can also capture unbound Tl^+^ within the intracellular environment.

### Radiotoxicity of ^201^Tl-caPBNPs and ^201^Tl-chPBNPs in Lung Cancer Cells

The radiotoxicity of radiolabeled caPBNPs and chPBNPs was assessed by performing clonogenic assays to measure the long-term survival and reproductive capacity of the treated cells and γH2AX assays to measure the relative frequency of DNA double strand breaks (DSBs), denoted as the average number of foci per nucleus.

To eliminate any potential cytotoxic effects caused either by PBNPs or the coatings used for the PBNPs, clonogenic assays after incubation of cells with 0.012 to 0.4 mg/mL of unlabeled, nonradioactive nanoparticles were performed (Figure S2). CaPBNPs did not cause any cytotoxicity in concentrations in medium up to 0.4 mg/mL (Figure S2A), whereas chPBNPs showed some cytotoxicity, reducing clonogenic survival to 43.7 ± 6.0% at a concentration of 0.1 mg/mL (Figure S2B). The concentration of chPBNPs was therefore kept at 0.05 mg/mL in in vitro radiobiological experiments and in vivo experiments with ^201^Tl.

**Figure 3:**
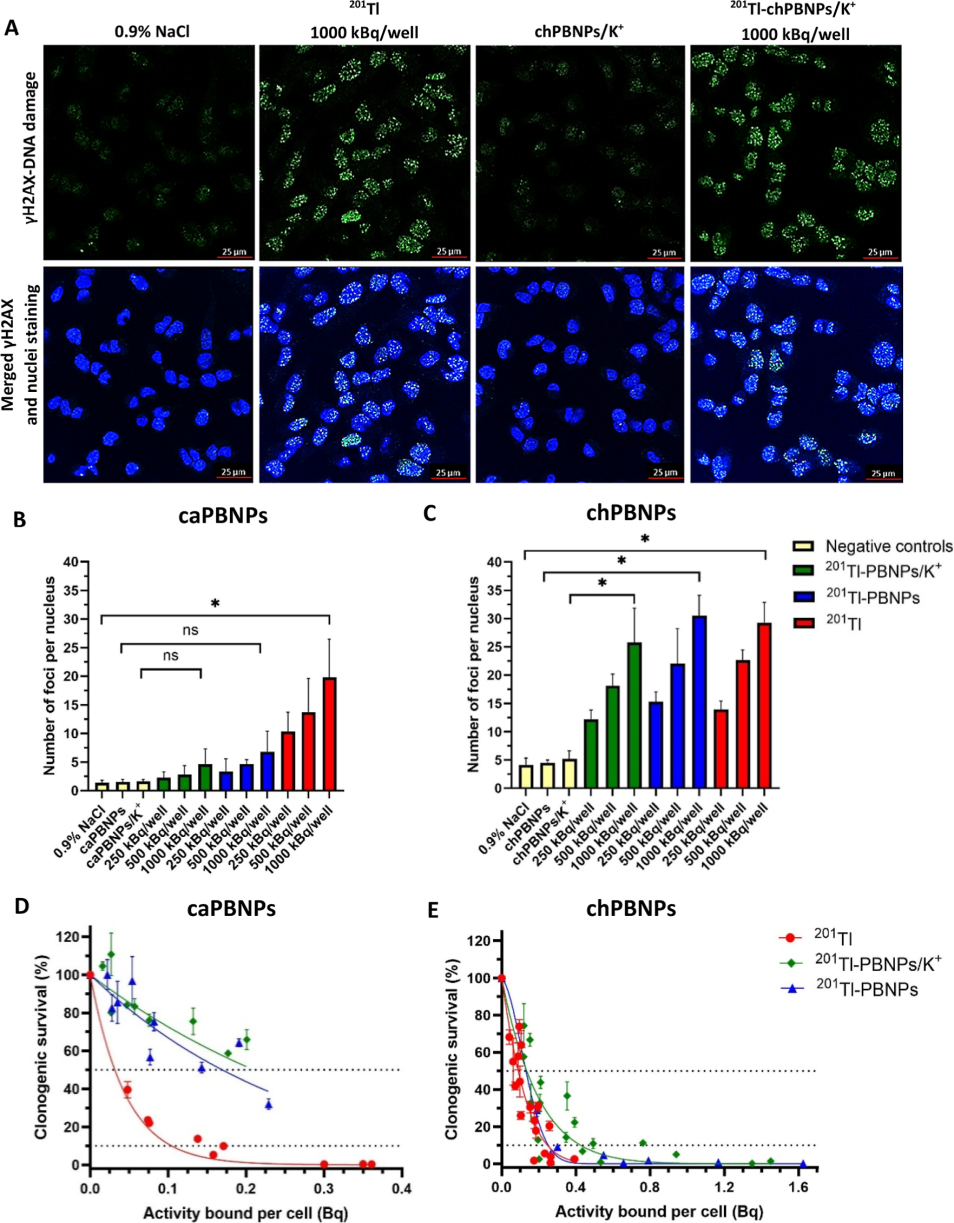
Radiotoxicity of ^201^Tl-caPBNPs and ^201^Tl-chPBNPs in lung cancer cells. (A) confocal microscopy images (100×, scale bar = 25 μm) of A549 lung cancer cells incubated for 3 h with 0.9% NaCl (1st negative control, 1st column), [^201^Tl]TlCl (2nd column), unlabeled chPBNPs/K^+^ (2nd negative control, 3rd column), and ^201^Tl-chPBNPs/K^+^ (4th column), followed by immunofluorescence staining with green fluorescence for γH2AX (1st row). Nuclear DNA is stained with DAPI (blue, 2nd row). (B) average number of foci per nucleus in A549 cells after 3 h incubation with varying activities of [^201^Tl]TlCl or ^201^Tl-caPBNPs with and without added KCl, compared to controls incubated with nonlabeled caPBNPs with and without added KCl and untreated controls (0.9% NaCl). (C) average number of foci per nucleus in A549 cells after 3 h incubation with varying activities of [^201^Tl]TlCl, ^201^Tl-chPBNPs with and without added KCl, compared to controls incubated with nonlabeled chPBNPs with and without added KCl and 0.9% NaCl. Activity added per well ranged from 250–1000 kBq. (D) clonogenic survival (%) of A459 cells as a function of the average activity bound per single cell after 3 h incubation with [^201^Tl]TlCl, ^201^Tl-caPBNPs, or ^201^Tl-caPBNPs/K^+^ in A549 cells (*n* = 3). (E) clonogenic survival (%) of A459 cells as a function of the average activity bound per single cell after 3 h of incubation with [^201^Tl]TlCl or ^201^Tl-chPBNPs or ^201^Tl-chPBNPs/K^+^ (*n* = 3 or 6). Linear quadratic survival model was fitted, and activity causing at least 50% and 90% reduction was calculated for ^201^Tl-chPBNPs/K^+^, ^201^Tl-chPBNPs, and [^201^Tl]TlCl and is shown in Table 1. The concentration of caPBNPs in medium: 0.1 mg/mL; concentration of chPBNPs in medium: 0.05 mg/mL. Some error bars are smaller than the data points and not visible. Bars represent mean ± SD, *n* = 3 in all experiments except for ^201^Tl and ^201^Tl-chPBNPs/K^+^ in (E) where *n* = 6; * indicates *P* < 0.05, Mann–Whitney test.

Examples of γH2AX fluorescence microscopy images for A549 cells treated with ^201^Tl-caPBNPs, ^201^Tl-chPBNPs, and unbound ^201^Tl^+^ are presented in Figures 3A and Figures S3 and S4. Quantitative data from γH2AX experiments revealed that ^201^Tl-caPBNPs had nonsignificant effects on the frequency of detected DSBs at activities of 250–1000 kBq/well compared to untreated cells and cells incubated with unlabeled caPBNPs (Figure 3B). On the other hand, cells treated with 1000 kBq/well of ^201^Tl-chPBNPs had a significantly higher average number of foci per nucleus (30.5 ± 3.6) compared to untreated cells and cells incubated with unlabeled-chPBNPs (4.1 ± 1.2 and 4.5 ± 0.4, respectively; Figure 3C). Incubation with the same activity of unbound ^201^Tl^+^ induced an average of 29.3 ± 3.6 foci per nucleus, around 6.5-fold higher than in cells treated with unlabeled-chPBNPs (Figure 3C). Thus, ^201^Tl-chPBNPs caused similar DSB frequency comparable with that caused by unbound ^201^Tl^+^, and much higher than that caused by ^201^Tl-caPBNPs, when measured as a function of the activity added to the medium. This is qualitatively consistent with the relatively low uptake of ^201^Tl-caPBNPs.

Clonogenic assay results are shown in Figure S5. Unbound ^201^Tl^+^ was highly radiotoxic to A549 cells; treatment with 1000 kBq/well reduced clonogenic survival to 0.3 ± 0.1% of control levels (without KCl; Figure S5A). The same activity of ^201^Tl-caPBNPs was much less radiotoxic, but still significantly decreased clonogenic survival to 49.1 ± 0.4% (without KCl, Figure S5A). ^201^Tl-chPBNPs at a concentration of 1000 kBq/well showed a much higher level of radiotoxicity, reducing clonogenic survival by over 16-fold compared to unbound ^201^Tl^+^ (0.5 ± 0.3% and 8.3 ± 10.6%, respectively, Figure S5B). Addition of KCl afforded a high level of protection against the radiotoxic effects of unbound ^201^Tl^+^ (Figure S5B) and a more modest protection against both types of radiolabeled PBNPs (Figures S5A,B). Clonogenic survival rates for unbound ^201^Tl^+^ varied between experiments assessing the radiotoxicity of caPBNPs and chPBNPs, likely due to inherent differences in the tested cell lines such as variations between batches.

To determine whether the differences in clonogenic toxicity between the three forms of ^201^Tl were related to the different levels of uptake within cells (from Figures 1 and S1A,B), the clonogenic survival in the same experiments was plotted against the measured average levels of radioactivity taken up by the cells (average Bq/cell). The results are expressed in the form of survival curves in Figure 3D,E and in terms of parameters *A*_50_ and *A*_10_—the average activity per cell required to reduce clonogenicity to 50% and 10%, respectively, of the control value. The curves for both ^201^Tl-caPBNPs and ^201^Tl-chPBNPs were shifted above and to the right of those for unbound ^201^Tl^+^, showing that for the same average levels of internalized activity, the radiolabeled PBNPs were less radiotoxic than unbound ^201^Tl^+^. The intracellular activities needed to reduce the clonogenic survival by at least 50% (*A*_50_) or 90% (*A*_10_) for ^201^Tl-chPBNPs and ^201^Tl^+^ with and without additional potassium ions were extracted from Figure 3E and are shown in Table 1; the values for ^201^Tl-chPBNPs were similar to or higher than those for ^201^Tl^+^, thus, the higher clonogenic toxicity of ^201^Tl-chPBNPs than either ^201^Tl-caPBNPs or unbound ^201^Tl^+^ when measured as a function of activity available in the medium can be accounted for the increased uptake in cells and is not attributable to higher toxicity of internalized ^201^Tl-chPBNPs; indeed, although it was not possible to calculate the *A*_50_ or *A*_10_ for ^201^Tl-caPBNPs with confidence due to the data points being concentrated above 50% of the clonogenic survival (Figure 3D), it was clear from the survival curves that internalized radiolabeled PBNPs of both types were less radiotoxic than the same activity of ^201^Tl^+^.

**Table 1:** Clonogenic Radiotoxicity Calculations for Internalized ^201^Tl-chPBNPs and Unbound Tl^+^^a^

*A*_50_ (Bq/cell)			*A*_10_ (Bq/cell)		
^201^Tl	^201^Tl-chPBNPs	^201^Tl-chPBNPs/K^+^	^201^Tl	^201^Tl-chPBNPs	^201^Tl-chPBNPs/K^+^
0.09[0.07–0.11]	0.13[0.06–0.16]	0.14[0.09–0.18]	0.25[0.19–0.30]	0.25[0.22–0.29]	0.43[0.30–0.70]

a The table shows the average internalized activity per cell needed to reduce the clonogenic survival by at least 50% (*A*_50_) and at least 90% (*A*_10_) for ^201^Tl-chPBNPs in the presence and absence of KCl. Data are presented as mean ± SD. Confidence intervals of 95% (95% Cl) are shown in square brackets.

### Subcellular Localization of Tl-caPBNPs and Tl-chPBNPs in Lung Cancer Cells

**Figure 4:**
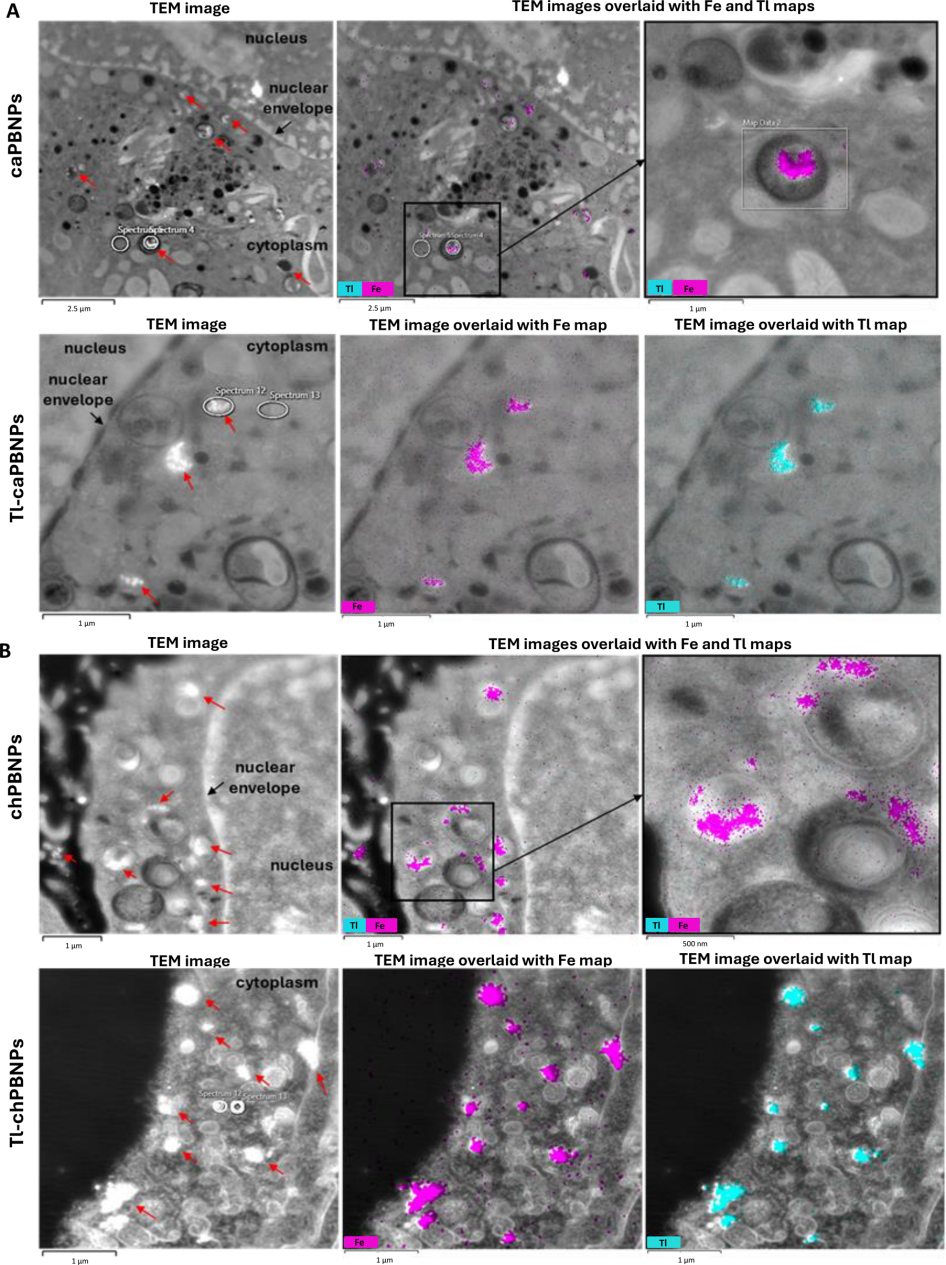
TEM/EDS analysis of caPBNPs, Tl-caPBNPs, chPBNPs, and Tl-chPBNPs in A549 lung cancer cells. (A) TEM images and overlays with iron (magenta) and thallium (turquoise) EDS signal of a thin cell section showing native thallium-free caPBNPs (first row) and thallium-doped caPBNPs (second row). (B) TEM images and overlays with the iron and thallium EDS signal of a thin cell section showing native thallium-free (first row) and thallium-doped chPBNPs (second row). Red arrows indicate PBNPs. The top right panels in both A and B show magnified regions of the respective center panels. PBNPs are visible here as white dots corresponding with the higher electron density.

TEM combined with energy-dispersive X-ray spectroscopy (EDS) was used to establish the subcellular localization of radiolabeled PBNPs in A549 cells. Both types of nanoparticles were detected in the cytoplasmic region of the cell, visible as white dots (indicating high electron density) in the TEM images, mostly clustered in vesicular compartments (Figure 4), but were not seen within the nuclear envelope. More chPBNPs than caPBNPs were seen within cells, consistent with the relative uptake levels shown in Figure 1, despite the lower concentration of the former used for sample preparation. EDS analysis confirmed the identity of these bright features as PBNPs by detecting the characteristic X-ray energy of iron emitted by the nanoparticles (Figure 4). Individual nanoparticles within the cytoplasm were also observed (Figures S6 and S7), but almost none were found in the nuclei of cells. Nanoparticles doped with stable thallium, but not thallium-free nanoparticles, also exhibited a thallium EDS signal colocalized with the iron signal and the TEM foci of the PBNPs (Figures 4, S8). Quantitative EDS spectra (Figure S8) showed an average total weight percentage (wt %) of thallium in regions rich in Tl-doped caPBNPs of 3.98 ± 1.55% compared to 0.03 ± 0.05% in nanoparticle-free areas (Figure S9). An even higher increase in wt % was noted for Tl-doped chPBNPs, from 0.04 ± 0.10% in nanoparticle-free regions to 6.47 ± 5.20% in nanoparticle-rich regions. By contrast, unbound thallium accumulated in cells was below the detection limit of EDS (∼0.1 wt %), suggesting a diffuse rather than focal distribution. This study showed that PBNPs after 3 h of incubation remained largely intact inside cells, with thallium remaining concentrated within the crystal structure of PBNPs, accumulating in specific subcellular regions, but not appreciably within nuclei.

### Comparative Biodistribution of ^201^Tl-chPBNPs and [^201^Tl]TlCl in Tumor-Bearing Mice

**Figure 5:**
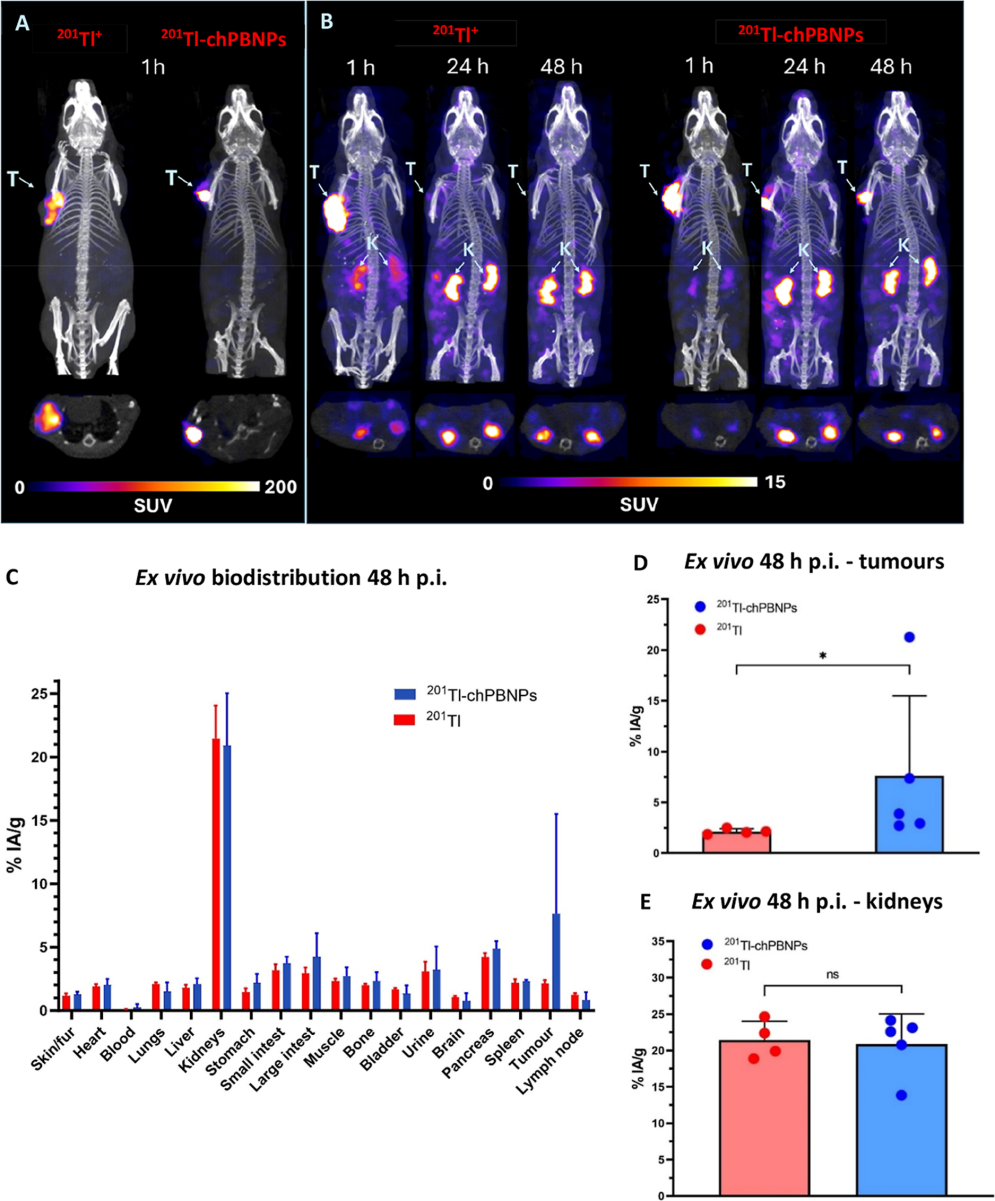
In vivo radioactivity distribution after 1, 24, and 48 h and ex vivo biodistribution at 48 h. (A) MIP images taken 1 h after injecting mice intratumorally with [^201^Tl]TlCl (left) and ^201^Tl-chPBNPs (right) and associated transverse images showing the activity distribution within the tumor (SUV scale: 0–200). (B) MIP images taken 1, 24, and 48 h after injecting mice with [^201^Tl]TlCl (three to the left) and ^201^Tl-chPBNPs (three to the right). The associated transverse images show the activity accumulated in kidneys (SUV scale: 0–15, is more sensitive than in A in order to show activity distribution outside tumors). CT images are overlaid with SPECT images providing anatomical reference (grayscale). Arrows indicate tumors (T) and kidneys (K). (C) ex vivo biodistribution in organs 48 h after mice were intratumorally injected with [^201^Tl]TlCl (control group) and ^201^Tl-chPBNPs, presented as % IA/g. (D) ex vivo biodistribution in tumors (as % IA/g) 48 h after mice were intratumorally injected with [^201^Tl]TlCl and ^201^Tl-chPBNPs. (E) ex vivo biodistribution in kidneys (as % IA/g) 48 h after mice were injected with [^201^Tl]TlCl and ^201^Tl-chPBNPs. Mann–Whitney test (D) and unpaired *t*-test (E) were used to assess significance, with *P* < 0.05 regarded as statistically significant. Data are shown as average ± SD (*n* = 4–5 mice per group), p.i.—postinjection.

To compare the retention of ^201^Tl-chPBNPs with that of unbound ^201^Tl^+^ in a subcutaneous murine xenograft model, A549 tumor-bearing mice were injected intratumorally with 0.4–0.5 MBq of [^201^Tl]TlCl (control group) or ^201^Tl-chPBNPs. SPECT/CT images acquired after administration showed that in both groups, by 1 h postinjection (p.i.), most activity was contained within the tumors (Figure 5A) with a very small amount of activity in the kidneys (Figure 5B). However, SPECT imaging at later time points revealed slower efflux from tumors in the mice injected with ^201^Tl-chPBNPs, with 2.1 ± 1.3% IA remaining in tumors at 24 h compared to 0.4 ± 0.1% IA for mice injected with [^201^Tl]TlCl (*n* = 3, *p* = 0.09, Figures 5B, S10A, S11). At 48 h, images showed 1.4 ± 1.0% IA remaining in tumors in the ^201^Tl-chPBNPs group compared to just 0.3 ± 0.1% IA in the [^201^Tl]TlCl group (*n* = 3, *p* = 0.1, Figure S10A). The remaining activity was predominantly detected in kidneys, with smaller amounts distributed across other organs such as small and large intestine (Figures 5B, S10B, and S11).

The biodistribution trends observed by SPECT imaging were placed on a quantitative footing by post-mortem ex vivo organ counting. Radioactivity concentrations (% IA/g) in tissues are shown in Figure 5C, confirming that ^201^Tl administered as ^201^Tl-chPBNPs showed retention in tumors was significantly (around 3.6 fold) higher than unbound ^201^Tl^+^ (7.6 ± 7.0% IA/g and 2.1 ± 0.3% IA/g, respectively, *p* = 0.02; Figure 5D). Outside the tumors, the biodistribution did not differ significantly between the two groups (Figure 5C); ^201^Tl activity was mostly present in kidneys, with an average of 21.5 ± 2.6% IA/g in the control group and 20.9 ± 4.1% IA/g in the ^201^Tl-chPBNPs group (Figure 5E). Activity was also present in urine, indicating predominantly renal excretion in both groups. Figure S12 presents the biodistribution in both groups expressed as % IA. Overall, the in vivo biodistribution experiments showed that after intratumoral injection in mice, radioactivity administered in the form of ^201^Tl-chPBNPs was retained within the tumors significantly longer than unbound ^201^Tl^+^.

## Discussion

The in vitro assessment of the cellular behavior of radiolabeled caPBNPs and chPBNPs consisted of uptake assays, efflux assays, and two different radiotoxicity assays aiming to compare the radiotoxic potential of ^201^Tl bound by nanoparticles with that of unbound ^201^Tl^+^. While ^201^Tl bound to the negatively-charged caPBNPs was taken up by cancer cells only in small amounts (lower than unbound ^201^Tl^+^), we observed significantly higher uptake of ^201^Tl bound to the positively-charged chPBNPs in all tested cell lines, compared to unbound ^201^Tl^+^. This could be explained by the different surface charge of the nanoparticles: positively charged nanoparticles often exhibit enhanced interactions with the negatively charged cellular membrane due to electrostatic attraction, facilitating endocytosis leading to increased cellular uptake.^34^ Other factors, including nanoparticle size, shape, surface chemistry, and the specific type of cell are also known to influence nanoparticle uptake.^35^ Moreover, the amount of nanoparticle-bound radioactivity taken up by A549 cells steadily and continuously increased over time, without plateau, for both types of nanoparticles, whereas uptake of unbound ^201^Tl^+^ quickly reached a plateau (Figure S1A and S1B), indicating a dynamic equilibrium regulated by the relative rates of influx and efflux. This indicates that nanoparticle uptake is likely linked to endocytic mechanisms limited only by the availability in medium, differing from the uptake of unbound thallium ions, which is reversible over a short time scale and mimics, and is limited by, the *trans*-membrane potassium gradient.^36,37^

The ability of PBNPs to capture thallium when they are already located inside cells was also tested. In A549 cells without preloading of nanoparticles, unbound Tl^+^ uptake, driven largely by the Na^+^/K^+^-ATPase pump, reached around 12% uptake, corresponding to its typical equilibrium intracellular-to-extracellular concentration ratio of 32:1 (very similar to literature estimates for K^+^ ratios ranging from 30:1 to 50:1)^38^ after 90 min. In cells preloaded with PBNPs, the ^201^Tl uptake by 90 min was significantly higher, reaching almost 40% for caPBNPs, corresponding to an intracellular-to-extracellular ratio of 104:1 (with an initial concentration in medium of 0.2 mg/mL; more than 3-fold higher than untreated cells) and 65% for chPBNPs, corresponding to an intracellular-to-extracellular ratio of 261:1 (with an initial concentration in medium of 0.05 mg/mL; more than 8 fold higher than untreated cells). These findings suggest that PBNPs are able to bind thallium ions inside cells and effectively sequester them from cellular cytoplasm, resulting in further compensating influx of thallium ions from the medium to reach a new thermodynamic equilibrium. This increased ^201^Tl uptake in cells may lead to higher radiotoxicity and therefore could increase the efficiency of potential therapy using ^201^Tl. This approach, a form of pretargeting, has been applied in targeted therapies where the targeting vehicle and the radiotoxic agent are administered separately in order to enhance the therapeutic effect while reducing possible side effects.^39,40^ Pretargeting has not yet been studied for radioactive thallium; nor has the radiotoxicity of ^201^Tl when cells have been preincubated with PBNPs and it warrants further investigation. Conversely, it is also possible that the sequestration of Tl^+^ by PBNPs within cancer cells may have the potential to hinder the accumulation of intracellular ^201^Tl in the most sensitive subcellular targets, such as the cell nucleus, potentially leading to a reduction in its toxicity compared with the same activity of unbound ^201^Tl. As discussed below, our subcellular distribution measurements and radiotoxicity experiments address this issue.

The influence of PBNPs on the rate of ^201^Tl release from cells could also impact the utility of these nanoparticles in radionuclide therapy. Unbound ^201^Tl^+^ washes out from A549 cells rapidly when the media are replaced by fresh ^201^Tl-free media, with less than 10 min required to remove 50% of the initially accumulated activity. After repeated changes of surrounding medium, mimicking the physiological environment of continuously perfused tumor cells, nearly all of the unbound thallium is washed out. The washout rate, however, slows down in the presence of PBNPs: more than 20 min for caPBNPs and nearly 1 h for chPBNPs were required to wash out 50% of the initial radioactivity, and significant activity levels remained within cancer cells even after multiple medium replacements; 23.4% for caPBNPs and 39.3% for chPBNPs, compared to almost none without PBNPs (Figure S1). This was evident both after the conventional uptake (Figure 1) and after the pretargeted uptake (Figure 2). Considering the long half-life of ^201^Tl (73 h), the delayed efflux highlights the potential of these nanoparticles to enhance the efficacy of ^201^Tl radionuclide therapy. However, in this scenario, again, we should also consider the differences in subcellular distribution between ^201^Tl bound to PBNPs and unbound ^201^Tl^+^.

TEM/EDS was able to localize unlabeled PBNPs and associated thallium in lung cancer cells, and around 130 to 160 times higher thallium concentration is present in the PB-rich subcellular regions compared to the nanoparticle-free regions, indicating that intracellular thallium remains mainly bound to the PBNPs; any thallium that is released from the PBNPs while inside the cell is likely to quickly escape from the cell, presumably in the form of Tl^+^ ions. The finding that both types of PBNPs were predominantly observed in clusters in vesicular compartments in the cytoplasmic region of the cell, and were almost absent from cell nuclei, matches previous observations of caPBNPs being contained within vesicles in the cytoplasm in breast cancer cells^23^ and is likely to have significant implications for the radiotoxicity of ^201^Tl-PBNPs. The differing subcellular localization of PBNP-bound Tl and unbound Tl^+^ may explain the variations in the observed radiobiological effects. The lack of significant PBNP accumulation within the nuclear envelope suggests that, while appropriately modified PBNPs (e.g., chitosan) can enhance the overall cellular uptake of ^201^Tl compared to unbound ^201^Tl, they may also provide protection against its radiotoxicity. This protective effect could arise from reducing irradiation to sensitive subcellular targets, such as nuclear DNA, by mitigating exposure to ^201^Tl and its very short-range electron emissions. However, this is uncertain, as it is still unknown whether unbound ^201^Tl^+^ ions can accumulate in the cell nucleus. Unbound Tl^+^ was not detected in cells using the EDS/TEM method, likely because its distribution was diffuse rather than punctate and hence did not reach a detectable concentration within any cell compartment. Further research should delve deeper into the subcellular localization of unbound Tl^+^ and investigate strategies to direct PBNP-bound thallium not only to specific cancer receptors but also to radiosensitive subcellular targets.

As a function of the amount of radioactivity added to the media surrounding A549 cells, ^201^Tl-caPBNPs were significantly less effective at decreasing clonogenic survival than the same activity of unbound ^201^Tl^+^ (Figure S5). This is likely mainly because ^201^Tl-caPBNPs delivered a smaller fraction of administered radioactivity into the cells (by a factor of about 0.75, see Figure 1A) than unbound ^201^Tl^+^. This is consistent with the observation that DNA damage foci, detected by the γH2AX assays, were significantly less frequent when ^201^Tl was delivered in the form of ^201^Tl-caPBNPs than the same activity delivered as unbound ^201^Tl^+^ (Figures S3 and 3B); presumably, again, because less of the administered radioactivity was accumulated in the cells. The positively charged ^201^Tl-chPBNPs, on the other hand, caused much more frequent DNA damage foci than ^201^Tl-caPBNPs, comparable with the same administered activity of unbound ^201^Tl^+^; however, since we have shown that the uptake of ^201^Tl-chPBNPs into A549 cells is significantly greater than that of unbound ^201^Tl^+^, it is anomalous that the level of DNA damage caused by ^201^Tl-chPBNPs was not even higher, as discussed below. The clonogenic toxicity measurements, to an extent, paralleled the γH2AX assays in that ^201^Tl-caPBNPs showed lower toxicity than the same administered activity of either unbound ^201^Tl^+^ or ^201^Tl-chPBNPs. Since our previous studies demonstrated that ^201^Tl must be delivered inside cancer cells to induce significant radiotoxicity,^6^ this could be accounted for by the much-reduced uptake of ^201^Tl-caPBNPs in cells. ^201^Tl-chPBNPs also showed higher toxicity than the same administered activity of unbound ^201^Tl^+^, consistent with their higher cellular uptake.

To determine whether this could be accounted for by the higher uptake of ^201^Tl-chPBNPs, we sought to take into account the difference in uptake efficiency between ^201^Tl-caPBNPs and unbound ^201^Tl^+^ by also measuring clonogenic toxicity as a function of the average accumulated activity per cell during the 3 h incubation period, rather than merely the administered activity, expressed in the form of survival curves (Figure 3D,E) and in the form of the *A*_50_ and *A*_10_ parameters (Table 1). In the case of ^201^Tl-caPBNPs, *A*_50_ and *A*_10_ could not reliably be computed, because the activities used in the experiment did not cause sufficient toxicity. Nevertheless, it is readily apparent from the form of the curve shown in Figure 3D that, on this accumulated activity-per-cell basis (i.e., even after correction for their reduced uptake of radioactivity), ^201^Tl-caPBNPs showed much less clonogenic radiotoxicity than unbound ^201^Tl^+^, and the clonogenic radiotoxicity of ^201^Tl-chPBNPs was reduced to levels comparable to or slightly less than that of ^201^Tl^+^ (Figure 3E and Table 1). This suggests that once inside the cell, sequestration of ^201^Tl within nanoparticles affords a degree of protection against radiotoxicity that would otherwise have been caused by unbound ^201^Tl^+^.

However, this analysis so far neglects the very different efflux behavior of the two tracers evident in Figure 1, which was not corrected for in the experimental design: whereas at the end of the incubation period of 3 h, unbound ^201^Tl^+^ is quickly washed out of cells, so that during the several days for which the cells were subsequently cultured as part of the clonogenic assay, the cells received little or no additional radiation dose. In contrast, ^201^Tl-chPBNPs washed out much more slowly and far from completely, and significant amounts would have remained within the cells during the clonogenic incubation period and thus imparted a large additional radiation dose. Although we did not measure the radioactivity retained by the cells during the course of the clonogenic assays, considering the efflux data shown in Figure 1 and the relatively long half-life of ^201^Tl, we suggest that the cells treated with ^201^Tl-PBNPs would have received a significantly higher cumulated radiation dose than cells treated with unbound ^201^Tl^+^. That this higher radiation dose did not elicit greater clonogenic radiotoxicity is further evidence that sequestration of ^201^Tl within the nanoparticles reduces the radiotoxicity of internalized ^201^Tl, possibly by preventing uptake in the nucleus (or other radiosensitive organelles). These observations point to the conclusion that while using ^201^Tl-chPBNPs as a delivery vehicle can enhance radiotoxicity compared to unbound ^201^Tl^+^ by increasing uptake of radioactivity in the cell and prolonging its retention to take full advantage of the long half-life, this gain is to an extent mitigated by the reduction in radiotoxic effect (both in terms of clonogenic survival and frequency of DNA damage foci) per Becquerel of internalized ^201^Tl. This is most likely caused by differences in subcellular distribution, considering the short-range of the emitted Auger electrons. One might surmise that it is due to the prevention of radioactivity from reaching a radiosensitive cellular compartment, such as the nucleus, since chPBNPs were not seen in significant numbers in cell nuclei in the TEM and EDS experiments. The net gain in therapeutic potential is dependent on specific circumstances that control the balance of these factors.

Although intracellular ^201^Tl-chPBNPs were less radiotoxic than intracellular unbound ^201^Tl^+^ in our in vitro experiments, we demonstrated that ^201^Tl^+^ can be efficiently encapsulated by the crystal structure of PBNPs and successfully delivered into cancer cells, resulting in an increased retention time. If we assume that the specific subcellular localization contributed significantly to the observed reduction in radiotoxicity, further modifications to chPBNPs structure, such as attaching nucleus-targeting peptides, may enhance nuclear uptake and potentially increase their toxicity.

Since ^201^Tl-chPBNPs afforded both increased cellular uptake and prolonged cellular retention of radioactivity in A549 cells compared to unbound ^201^Tl^+^, we sought to determine whether this effect persists in vivo. Therefore, these nanoparticles were studied in vivo in a subcutaneous A549 tumor in a group of mice to assess the retention of activity within the tumor tissue, compared to a second group injected with unbound ^201^Tl^+^. Intratumoral delivery was chosen to circumvent the absence of tumor targeting in the current design of the particles and to prevent the potential sequestration by macrophages in liver, spleen, and bone marrow (i.e., reticuloendothelial uptake) that is typical of i.v.-administered nanoparticles. There are no previous in vivo studies investigating ^201^Tl-PBNPs after intratumoral injection and very few describing biodistribution of i.v.-administered ^201^Tl-PBNPs. One study with i.v.-injected dextran-coated PBNPs showed early (1–24 h) accumulation in the lungs and liver with later (48 h) accumulation in kidneys.^31^ Another study with ^201^Tl-PBNPs coated with glucose-functionalized aminotriethylene glycol or amino polyethylene glycol (average diameter: 2.4 ± 0.6 nm) found radioactivity predominantly in the kidneys and liver after 48 h.^29^ Both studies emphasized the significant impact of surface functionalization, nanoparticle size, and morphology on early biodistribution.

In the present study, efflux of ^201^Tl from the tumor was slower in the ^201^Tl-chPBNPs group, but the effluxed activity exhibited a similar pattern to the control group, with clearance primarily via the kidneys. The difference in tumor retention became more apparent at 24 and 48 h. The retention enhancement afforded by using PBNPs as carriers is consistent with the delayed efflux from cells seen in the in vitro experiments. The similarity in the biodistribution of activity outside the tumor in both groups, and the absence of a reticuloendothelial uptake pattern, suggests that release of ^201^Tl^+^ from ^201^Tl-chPBNPs in the tumor, followed by efflux of the released ^201^Tl^+^ from the tumor, is the main route of clearance, rather than the alternative possibility that the ^201^Tl-chPBNPs are released from the tumor intact or in smaller fragments. The latter possibility cannot be completely dismissed; however, PBNP pharmacokinetics are known to be controlled by their size and surface charge;^41^ smaller nanoparticles (less than 5 nm in diameter) are more likely to be excreted by the kidneys^42^ and interaction between the positively charged PBNPs, and blood proteins can further accelerate the elimination of nanomaterials in vivo.^42^

Tl^+^ that leaks from the PBNPs and subsequently exits the cells (as seen in the in vitro efflux experiments) could potentially undergo redistribution within the cell to give a more cytotoxic distribution in more radiosensitive organelles before leaving the cell, after which it could be recaptured by the same cell or neighboring cells via the Na^+^/K^+^-ATPase pump, before finally washing out from the tumor via blood. The opportunity for subcellular redistribution could offer therapeutic advantages compared to ^201^Tl delivered in the form of stable chelate conjugates, which might not be easily redistributable.

PBNPs are widely recognized for their outstanding safety profile and biocompatibility, making them highly suitable for a range of biomedical applications.^22−28^ Since its FDA approval under the name Radiogardase, PB has been effectively used in clinical settings to treat patients following radioactive exposure. In previous preclinical studies, PBNPs have demonstrated excellent serum stability, negligible cytotoxicity in healthy and tumor cells, and no histological changes after extended observation in major organs of mice such as heart or liver.^13,23,32,41,43−45^ The metabolic pathways of PBNPs are determined by their size and surface charge, with smaller nanoparticles being rapidly metabolized and excreted through the kidneys and liver, while larger nanoparticles are processed more slowly.^41^ Mouse studies demonstrate that PBNPs, regardless of their size, are efficiently cleared within 2 weeks of administration, without causing significant long-term toxicity.^41^ Although no immunogenic effects have been reported, further studies are required to explore the interactions between surface-modified PBNPs and immune cells. Future research should focus on understanding the intracellular fate of PBNPs, their accumulation and clearance in biological systems, and addressing potential immunogenicity and antigenicity to improve their safety profile and efficacy in nanomedicine.

## Conclusions

Given the absence of the existing effective chelators for ^201^Tl, PB nanoparticles emerge as a novel alternative for delivering radioactive thallium to cancer cells for targeted radiotherapy. Different surface coatings (citric acid or chitosan in this study) can be used to regulate or enhance uptake in cells without affecting the particles’ capacity to carry ^201^Tl. Positively-charged chPBNPs showed much greater internalization into cells compared to negatively-charged caPBNPs. Furthermore, both types of PBNPs showed delayed efflux of ^201^Tl from cells, compared to unbound ^201^Tl^+^, offering cellular pharmacokinetics more closely matched to the half-life of ^201^Tl, and hence a higher absorbed radiation dose within the cell. Both types of PBNPs, once internalized, demonstrated the capability to increase cellular uptake and retention of subsequently administered ^201^Tl^+^, presumably by sequestering thallium that enters the cell via the Na^+^/K^+^-ATPase pump. The enhanced uptake of ^201^Tl-chPBNPs led to higher radiotoxicity (whether assessed as clonogenicity or DNA damage) compared to that of ^201^Tl-caPBNPs or unbound ^201^Tl^+^. However, at similar levels of accumulated activity (Bq/cell) and similar exposure times (and hence similar average cellular radiation doses), ^201^Tl-chPBNPs caused significantly less DNA damage than unbound ^201^Tl^+^, and when corrected for the additional exposure time during the clonogenic assay, ^201^Tl-chPBNPs showed a lower clonogenic toxicity than unbound ^201^Tl^+^. Thus, the gain in radiation dose and cytotoxicity due to enhanced accumulation and delayed efflux is mitigated in part by reduced cytotoxicity per unit of cellular radiation dose, likely due to a different subcellular distribution of the radioactivity and hence of the radiation dose (e.g., perhaps, prevention of uptake in the nucleus). ^201^Tl-chPBNPs exhibited significantly delayed efflux of ^201^Tl from tumors in vivo compared to unbound ^201^Tl^+^, with a distribution of radioactivity released from the tumor resembling that of ^201^Tl^+^ and showing predominantly renal clearance. With the current lack of satisfactory chelators for ^201^Tl, PBNPs present an alternative avenue for delivering radioactive thallium to cancer cells for therapeutic purposes, albeit additional work is required to optimize their biodistribution and further enhance specific accumulation and retention in tumors.

## Supporting Information Available

Additional experimental details and results, including ^201^Tl-caPBNP/^201^Tl-chPBNPs uptake and efflux timeline, cytotoxicity of nonradioactive caPBNP/chPBNPs, ^201^Tl-caPBNPs/^201^Tl-chPBNPs DNA damage, and clonogenic radiotoxicity, TEM of PBNPs, energy-dispersive X-ray spectroscopy combined with TEM of Tl-PBNPs, evaluation of ^201^Tl-chPBNP retention in vivo and ex vivo, and machine settings for preclinical SPECT scanning (PDF)
