# Supplementary material for: In Vitro and In Vivo Radiotoxicity and Biodistribution of Thallium-201 Delivered to Cancer Cells by Prussian Blue Nanoparticles
Source: ACS Appl Mater Interfaces. 2025 Feb 21;17(9):13577–91. doi: 10.1021/acsami.4c21700 (PMC11891825; doi:10.1021/acsami.4c21700)
Supplement: Supplementary file 1 — am4c21700_si_001.pdf [file am4c21700_si_001.pdf]

Supporting Information

In Vitro and In Vivo Radiotoxicity and Biodistribution of Thallium-201 Delivered to Cancer Cells by Prussian Blue Nanoparticles

Katarzyna M. Wulfmeier[a], Juan Pellico[a&], Pedro Machado[b,c], M. Alejandra Carbajal[b], Saskia E. Bakker[d], Rafael T. M. de Rosales[a], Kavitha Sunassee[a], Philip J. Blower[a], Vincenzo Abbate[e*#] and Samantha Y. A. Terry[a*#]

[a] School of Biomedical Engineering and Imaging Sciences, King's College London, London, SE1 7EH, United Kingdom

[b] Centre for Ultrastructural Imaging, King's College London, London, SE1 9RT, United Kingdom

[c] Oxford Instruments NanoAnalysis, High Wycombe, HP12 3SE, United Kingdom

[d] Advanced Bioimaging, University of Warwick, Coventry, CV4 7AL, United Kingdom

[e] Institute of Pharmaceutical Sciences, King's College London, London, SE1 9NH, United Kingdom

[*] Email: vincenzo.abbate@kcl.ac.uk

[*] Email: samantha.terry@kcl.ac.uk

[#] S.T and V.A contributed equally to this work

^{201}Tl-caPBNPs and ^{201}Tl-chPBNPs uptake timeline and efflux in lung cancer cells.

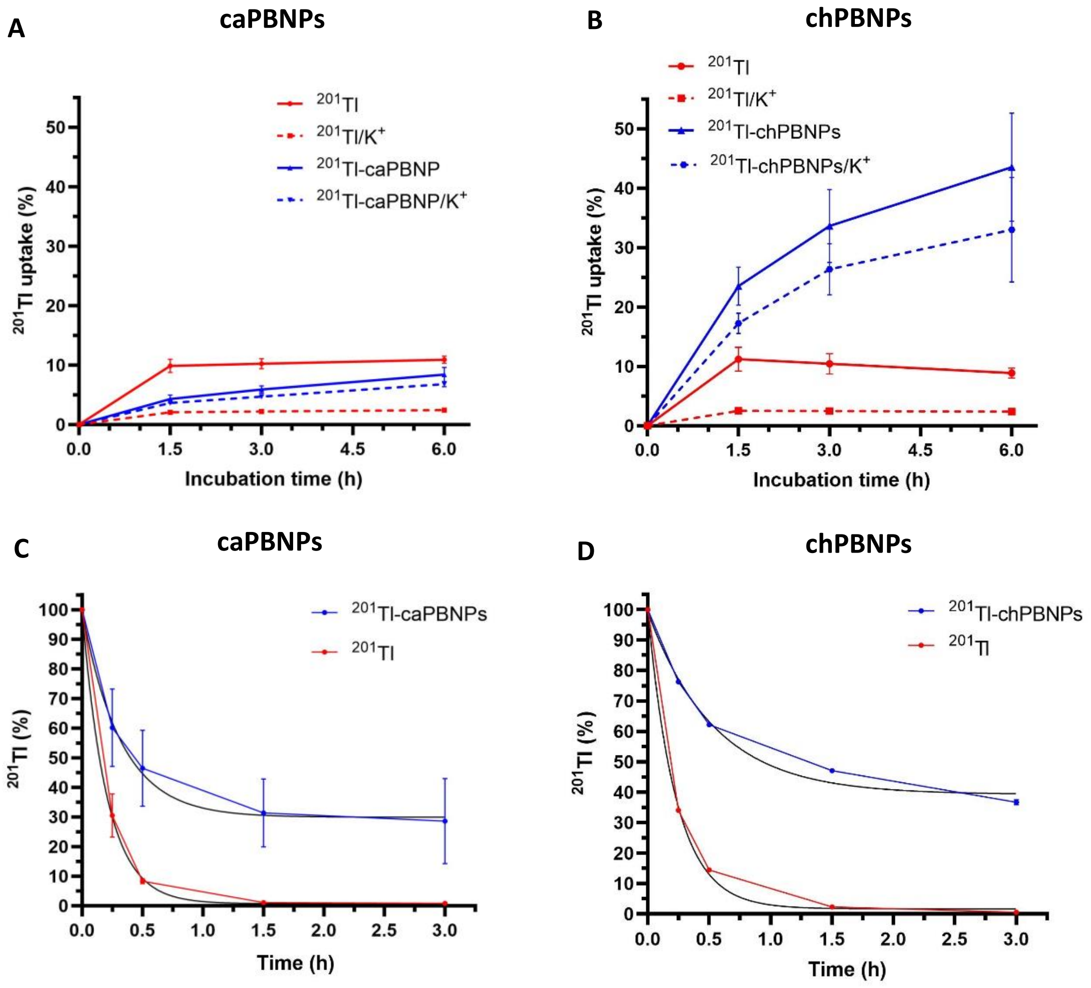

Figure S1. ^{201}Tl-PBNPs uptake timeline and efflux in lung cancer (A549) cells. A) Uptake timeline in A549 cells (up to 6 h) of $^{201}Tl^{+}$ and ^{201}Tl-caPBNPs. **B)** Uptake timeline in A549 cells (up to 6 h) of $^{201}Tl^{+}$ and ^{201}Tl-chPBNPs. 25 mM KCl was used to inhibit unbound ^{201}Tl uptake. **C)** Continuous efflux of ^{201}Tl and ^{201}Tl-caPBNPs vs time. The time needed to wash out 50% of the initial accumulated activity calculated for ^{201}Tl: 0.13 h (95% Cl: 0.13-0.14), plateau at 0.7% (95% Cl: 0-1.70); the time needed to wash out 50% of the initial accumulated activity calculated for ^{201}Tl-caPBNPs: 0.34 h (95% Cl: 0.20-0.56) plateau at 23.4%, (95% Cl: 6.50-38.0). **D)** Continuous efflux of $^{201}Tl^{+}$ and ^{201}Tl-chPBNPs vs time. The time needed to wash out 50% of the initial accumulated activity calculated for ^{201}Tl: 0.16 h (95% Cl: 0.14-0.19), plateau at 1.7% (95% Cl: 0-6.20); the time needed to wash out 50% of the initial accumulated activity calculated for ^{201}Tl-chPBNPs: 0.94 h (95% Cl: 0.17-1.33) plateau at 39.3% (95% Cl: 13.98-52.13). Data was fitted with an exponential decay equation (Y = (Y0 - plateau) *exp(-K*X) + plateau, black line, GraphPad Prism). Data are presented as mean ± SD, triplicates, n = 3 except C and D where n = 1. CaPBNP concentration: 0.1 mg/mL, chPBNPs concentration: 0.05 mg/mL. ^{201}Tl activity used: 30 kBq/well, 250,000 cells were seeded per well for each experiment.

Cytotoxicity of non-radioactive caPBNPs and chPBNPs in A549 lung cancer cells.

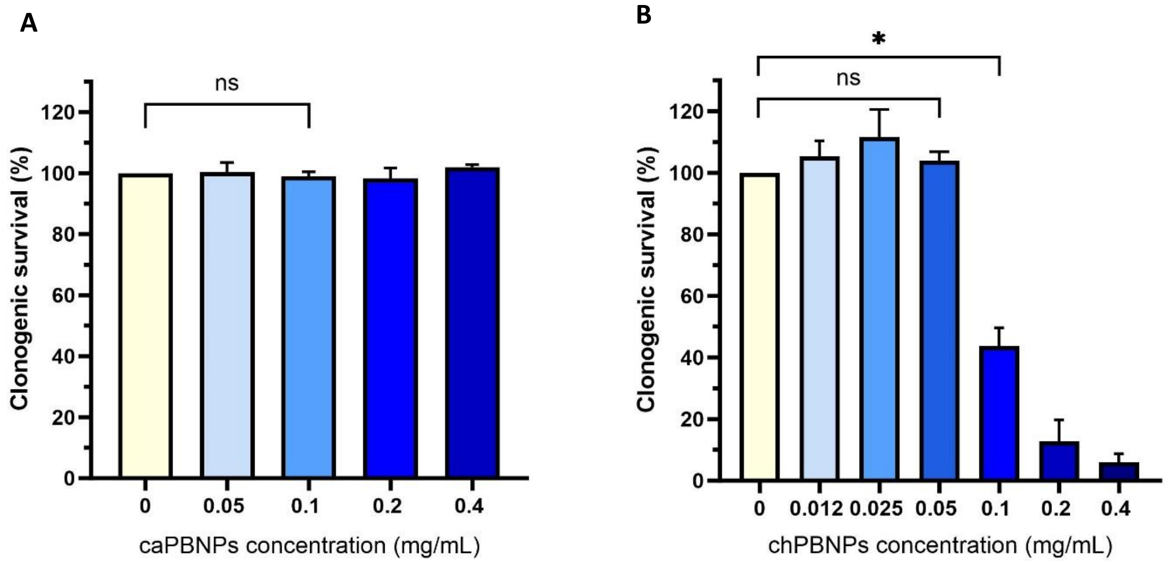

Figure S2. Cytotoxicity of non-radioactive caPBNPs and chPBNPs. A) caPBNPs cytotoxicity in lung cancer cells (A549) measured as clonogenic survivals (%) versus concentration in the medium (0.05 - 0.4 mg/mL). **B)** chPBNPs cytotoxicity in A549 cells measured as clonogenic survivals (%) versus concentration in the medium (0.012 - 0.4 mg/mL). chPBNPs concentrations higher than 0.05 mg/mL were not used in any of the *in vitro* or *in vivo* experiments. Data are presented as mean ± SD, triplicates, n = 3, * indicates $P < 0.05$, ns – not significant, paired t-test.

^{201}Tl-caPBNPs and ^{201}Tl-chPBNPs radiotoxicity in A549 lung cancer cells.

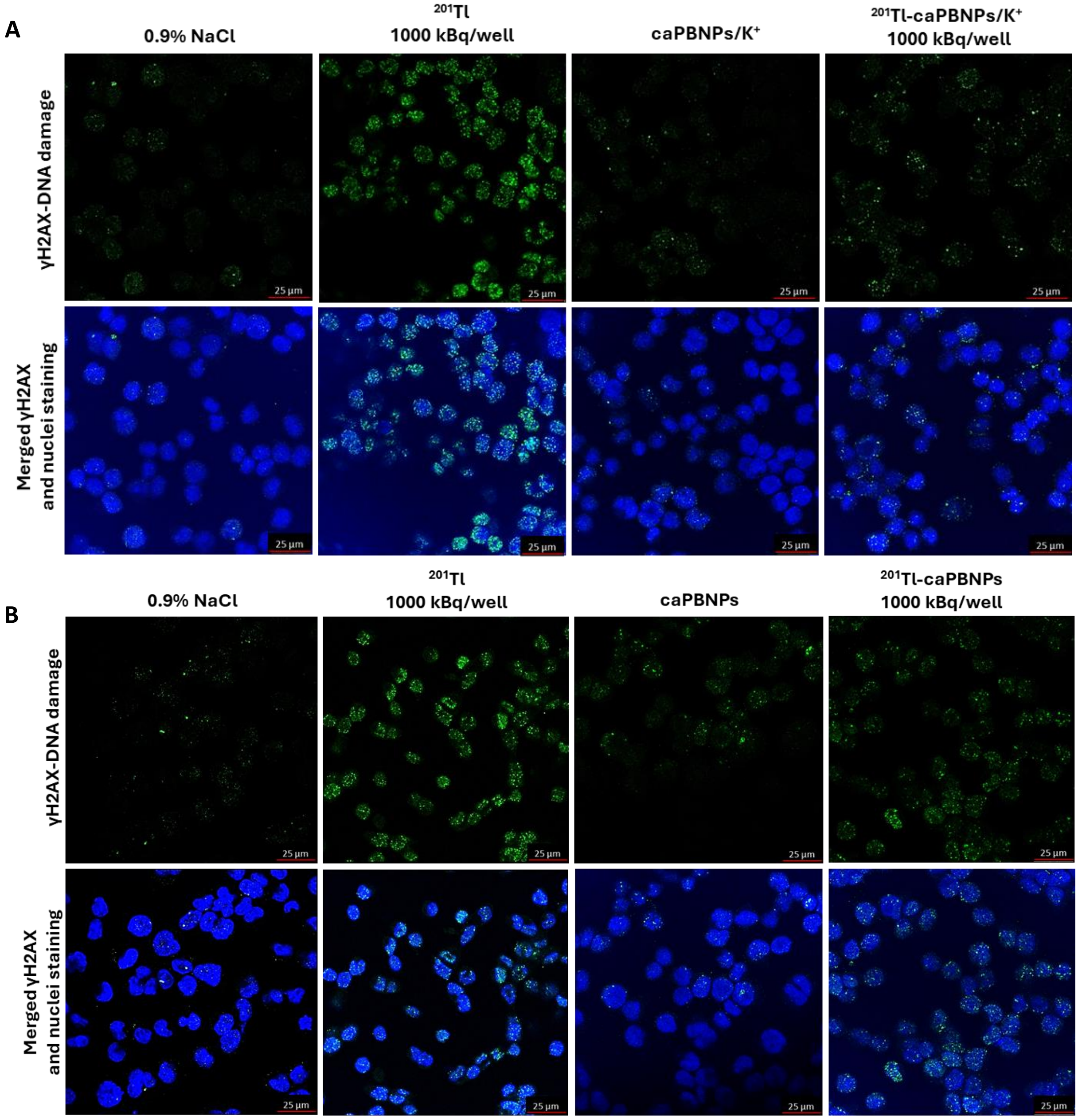

Figure S3. ^{201}Tl-caPBNPs nuclear DNA damage in lung cancer cells. Exemplar confocal fluorescence microscopy images (100x, scale bar - 25 µm) of lung cancer cells (A549) incubated for 3 h with **A)** 0.9% NaCl (negative control), [^{201}Tl]TlCl, caPBNPs with KCl (2nd negative control) and ^{201}Tl-caPBNPs with KCl; **B)** 0.9% NaCl (negative control), [^{201}Tl]TlCl, caPBNPs without KCl (2nd negative control) and ^{201}Tl-caPBNPs without KCl; followed by immunofluorescence staining with green fluorescence for γH2AX. Nuclear DNA is stained with DAPI (blue), ^{201}Tl^{+} activity: 1000 kBq/well.

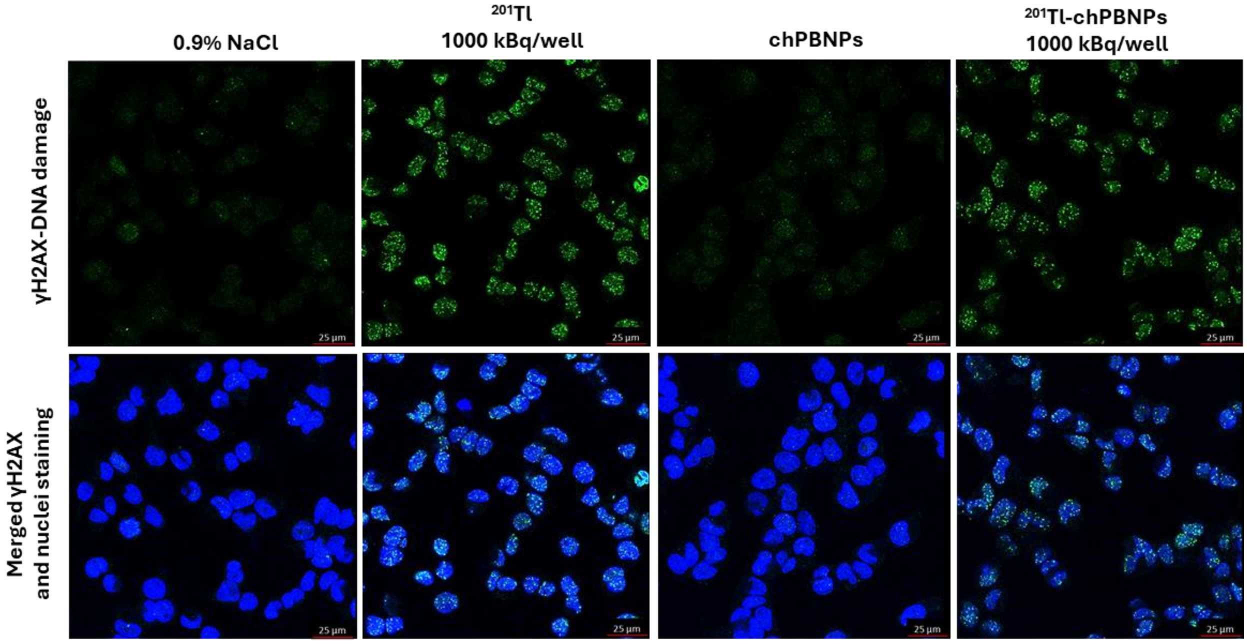

Figure S4. ^{201}Tl-chPBNPs nuclear DNA damage in lung cancer cells. Exemplar confocal fluorescence microscopy images (100x, scale bar - 25 µm) of lung cancer cells (A549) incubated for 3 h with 0.9% NaCl (negative control), [^{201}Tl]TlCl, chPBNPs without KCl (2nd negative control) and ^{201}Tl-chPBNPs without KCl; followed by immunofluorescence staining with green fluorescence for γH2AX. Nuclear DNA is stained with DAPI (blue), $^{201}Tl^{+}$ activity: 1000 kBq/well.

^{201}Tl-caPBNPs and ^{201}Tl-chPBNPs clonogenic radiotoxicity in A549 lung cancer cells.

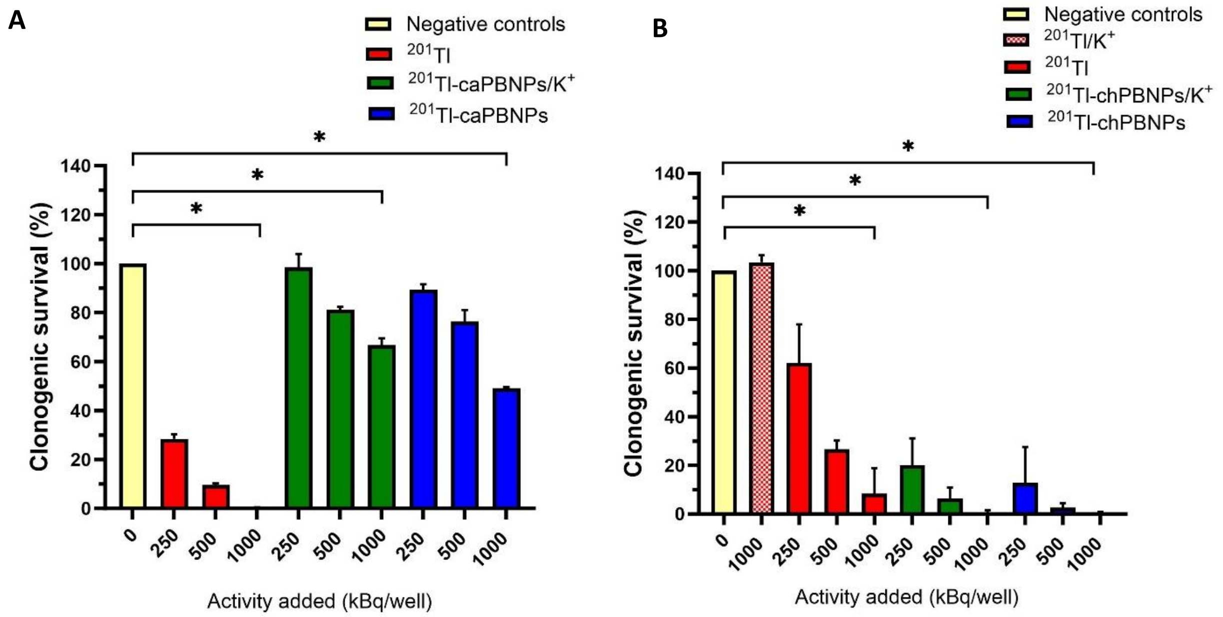

Figure S5. PBNPs clonogenic radiotoxicity in lung cancer cells. A) Clonogenic survival vs activity added per well in lung cancer cells (A549) treated with ^{201}Tl, ^{201}Tl-caPBNPs and ^{201}Tl-caPBNPs/K^{+}; caPBNPs concentration: 0.1 mg/ml. **B)** Clonogenic survival vs activity added per well in lung cancer cells (A549) treated with ^{201}Tl, ^{201}Tl-chPBNPs and ^{201}Tl-caPBNPs/K^{+}; chPBNPs concentration: 0.05 mg/ml. Data are presented as mean ± SD, triplicates, n = 3 or n = 6. Bars represent mean ± SD, * indicates $p < 0.05$, Mann-Whitney test. Clonogenic survival rates for unbound ^{201}Tl varied between experiments assessing the radiotoxicity of caPBNPs and chPBNPs, likely due to inherent differences in the tested cell lines, such as variations between batches.

Transmission electron microscopy (TEM) of PBNPs.

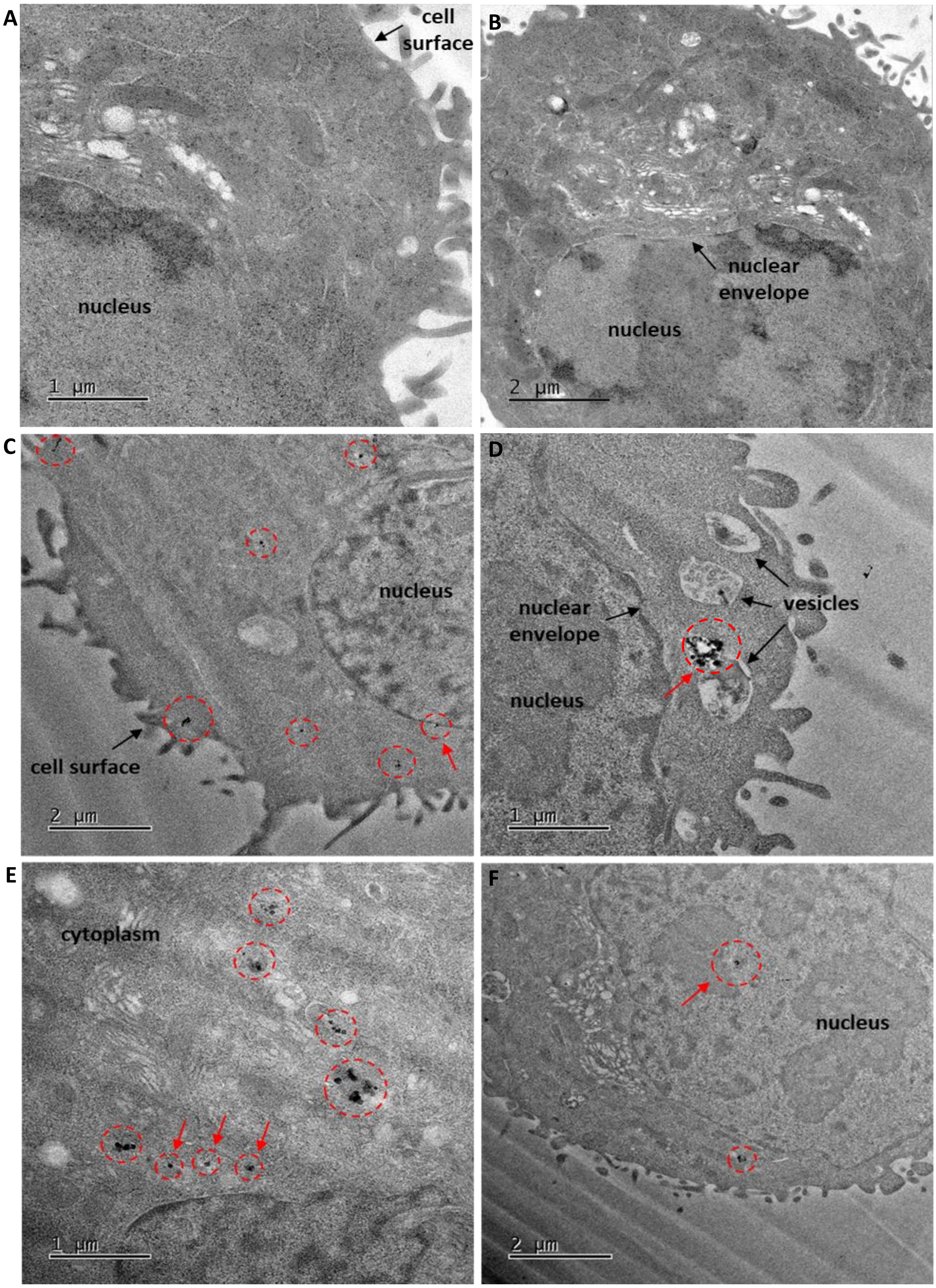

Figure S6. TEM images of caPBNPs in lung cancer cells. A) and **B)** TEM images of cell sections of lung cancer cells (A549) without PBNPs. **C), D), E)** and **F)** TEM images of cell sections of lung cancer cells (A549) incubated for 3 h with 50 µL of 0.5 mg/mL caPBNPs solution added to 200 µL RPMI medium. Cell nucleus, nuclear envelope, cytoplasm and cell surface are marked with black arrows. Nanoparticles visible are enclosed in red circles. Red arrows indicate caPBNPs interacting with the nuclear envelope (C), within a vesicle (D), individual caPBNPs (E) and caPBNPs in the cell nucleus (F).

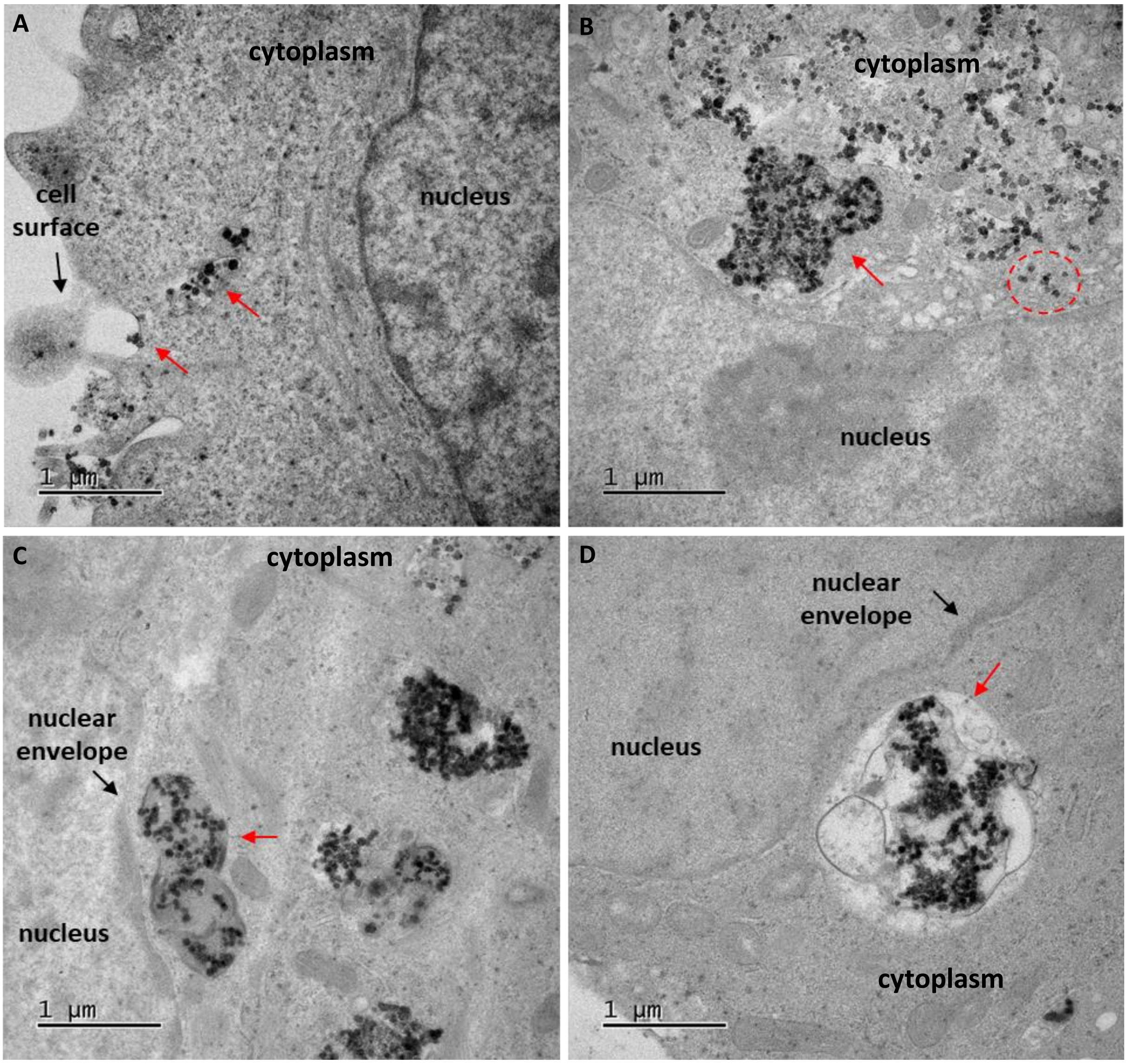

Figure S7. TEM images of chPBNPs in lung cancer cells. A), B), C) and **D)** TEM images of cell sections of lung cancer cells (A549) incubated for 3 h with 50 µL of 0.25 mg/mL chPBNPs solution added to 200 µL RPMI medium. Cell nucleus, nuclear envelope and cell surface are marked with black arrows. Individual nanoparticles are highlighted with a red circle (B). Red arrows indicate chPBNPs enclosed with the cell membrane (A) and chPBNPs within a vesicle close to the cell nucleus (B), (C) and (D).

TEM analysis for caPBNPs and chPBNPs was done at the Advanced Bioimaging Facility, University of Warwick. 250,000 lung cancer cells (A549) were seeded per well and 15 min before the experiment, the medium in each well was replaced by 200 µL fresh medium and 50 µL of MilliQ water, caPBNPs (final concentration: 0.1 mg/mL) or chPBNPs (final concentration: 0.05 mg/mL) was added into each well. After 3 h incubation time, medium was removed, cells were washed twice with cold PBS, trypsinised, moved to eppendorf tubes and centrifuged at 0.5 g for 5 min. Then the supernatant was removed and 800 µL of 2.5% EM grade glutaraldehyde solution (G015, TAAB) in PBS was added. After 1 h incubation at room temperature, cells were centrifuged at 0.5 g for 5 min, re-suspended in 500 µL PBS and centrifuged again. This process was repeated twice. After stepwise dehydration in 25%, 50%,

75% and 100% acetone, cells were infiltrated with 50% resin for 1 h followed by 100% resin (Agar Scientific Low Viscosity Epoxy Resin) for 24 h. The resin was left at 60°C overnight and then ultrathin sections on an RMC ultramicrotome were cut and stained in 2% uranyl acetate. The samples were imaged in a JEOL JEM 2100 Plus with Gatan OneView CMOS camera.

Transmission electron microscopy (TEM) combined with energy dispersive X-ray spectroscopy (EDS) of Tl-PBNPs.

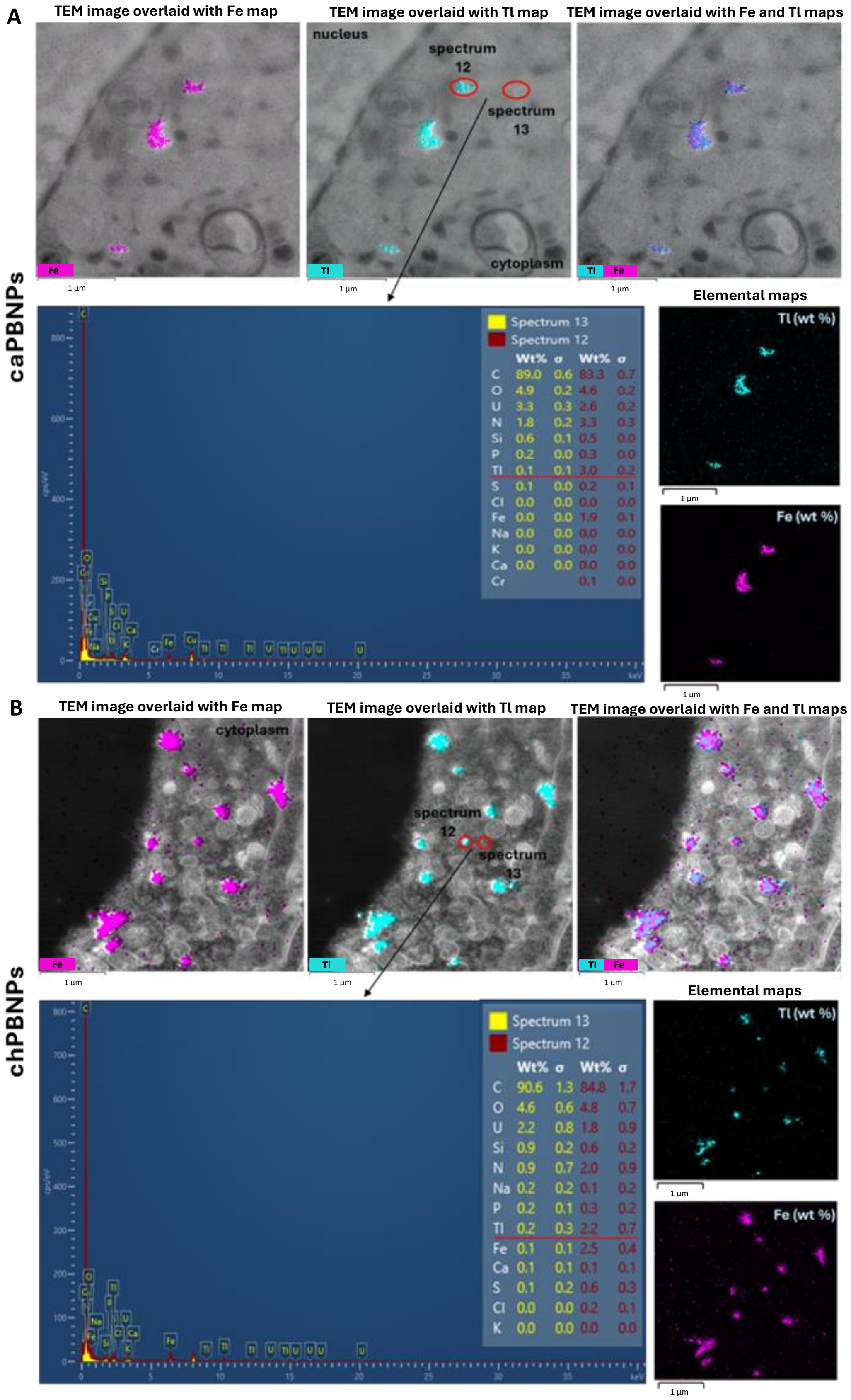

Figure S8. TEM/EDS analysis of Tl-caPBNPs and Tl-chPBNPs in lung cancer cells. TEM images of thin cell sections showing **A)** Tl-caPBNPs and **B)** Tl-chPBNPs overlays with iron and thallium EDS signal, energy spectrum comparing elemental composition in nanoparticle region (spectrum 12) and nanoparticle-free region (spectrum 13) and elemental maps of thallium and iron (expressed as % of total weight). The red circles indicate the regions of interest with and without Tl-caPBNPs and Tl-chPBNPs.

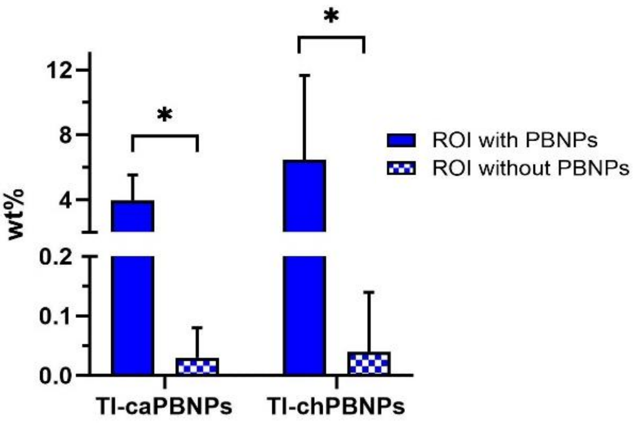

Figure S9. Quantitative EDS analysis of thallium bound to caPBNPs and chPBNPs. The quantification was based on energy spectra acquired from the regions of interest (ROIs) with and without Tl-caPBNPs and Tl-chPBNPs and is expressed as the average total weight percentage (wt%) of thallium. Bars represent mean ± SD, n = 10, * indicates $P < 0.05$, paired t-test.

Machine settings for pre-clinical SPECT scanning.

The NanoScan SPECT/CT scanner (80W, Mediso Ltd., Budapest, Hungary) was used for scanning mice. The standard mouse whole body collimator (APT63) with 64 pinholes was employed, and the acquisition was performed using Nucline^{TM} software version 3.04.025. The scanner was calibrated for ^{201}Tl before the experiment (activity calibration factor 1.61062). Three mice were scanned simultaneously using a 3-mouse hotel and counts were acquired using a dual energy window: 72.30 keV ± 10% and 167.40 keV ± 10%. SPECT image reconstruction was performed in Tera-TomoTM 3D SPECT reconstruction software using Regularised OSEM reconstruction with the following settings: TT3D high dynamic range, regularisation: medium, matrix size: 128x128, iterations: 48, subsets: 3, Monte Carlo quality: medium, attenuation and scatter correction and 0.5 mm isotropic voxel size. Subsequently, a helical CT acquisition was performed with 360 projections, pitch 1.0, 50 kVp, 980 mA, exposure time 170 ms, binning 1:4. The data were reconstructed in Nucline^{TM} version 3.04.025 using Filtered Back Projection (filter type: cosine) with isotropic voxel size of 0.25 mm. Decay correction was applied to time of injection. Mediso automated bed removal was applied using FusionTM software version 3.09.008 (Mediso).

Evaluation of ^{201}Tl-chPBNPs retention *in vivo*.

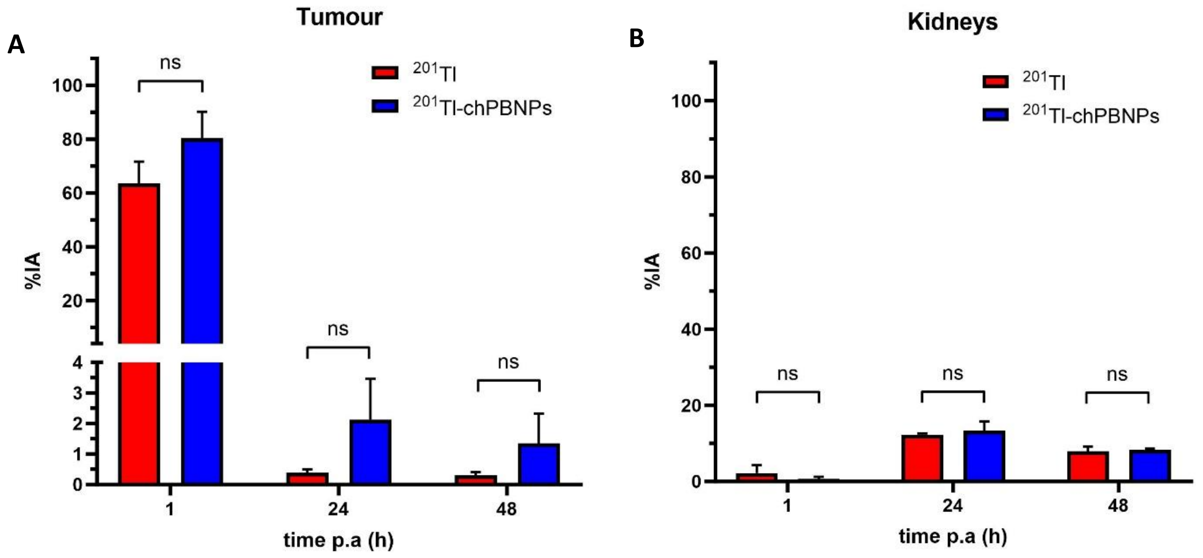

Figure S10. Quantitative analysis of SPECT/CT images after 1 h, 24 h and 48 h in mice injected with [^{201}Tl]TlCl and ^{201}Tl-chPBNPs in **A)** tumours and **B)** kidneys. Data is shown as average ± SD and expressed as % IA. n=3 mice per group. Data was tested for normal distribution using Shapiro-Wilk test; statistical significance was performed using unpaired t-test (non-significant), p.a - post-administration.

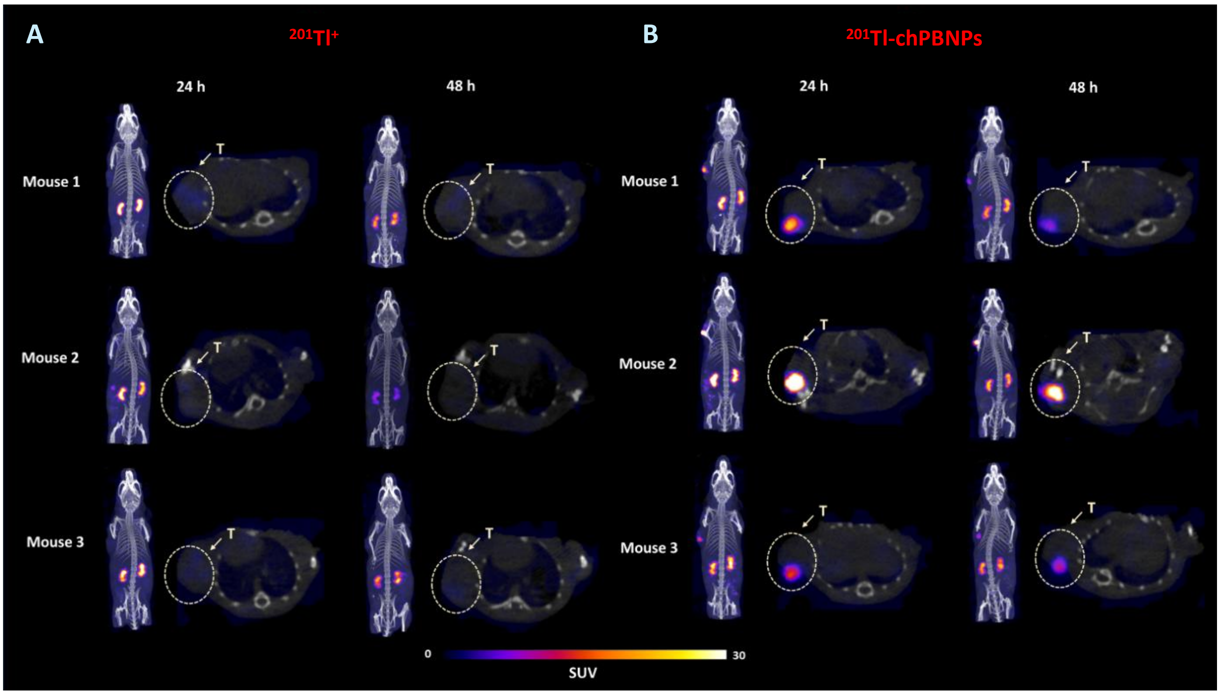

Figure S11. Activity distribution in tumours after 24 h and 48 h. MIPs and transverse images of SPECT/CT of mice injected with **A)** [^{201}Tl]TlCl (control group) and **B)** ^{201}Tl-chPBNPs after 24 h and 48 h post administration. The transverse images show the activity distribution within the tumour (white circle) with visibly higher activity present in mice injected with ^{201}Tl-chPBNPs compared to the control group. CT images are overlayed with SPECT images and used as anatomical reference (grayscale). SUV scale: 0-30. Arrows are pointing tumours (T).

Evaluation of ^{201}Tl-chPBNPs retention *ex vivo*

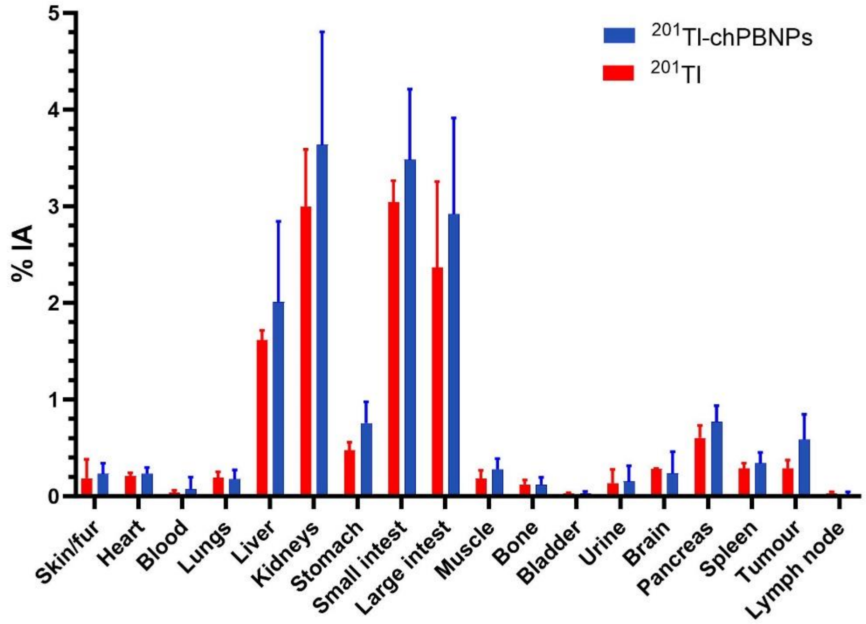

Figure S12. ***Ex vivo*** **biodistribution at 48 h presented as %IA** in mice injected with [^{201}Tl]TlCl (control group) and ^{201}Tl-chPBNPs). Data is shown as average ± SD, (n = 4 - 5 mice/group).
